# RNA-mediated control of cell shape modulates antibiotic resistance in *Vibrio cholerae*

**DOI:** 10.1038/s41467-020-19890-8

**Published:** 2020-11-27

**Authors:** Nikolai Peschek, Roman Herzog, Praveen K. Singh, Marcel Sprenger, Fabian Meyer, Kathrin S. Fröhlich, Luise Schröger, Marc Bramkamp, Knut Drescher, Kai Papenfort

**Affiliations:** 1 Institute of Microbiology, Friedrich Schiller University, 07745 Jena, Germany; 2 Faculty of Biology, Ludwig-Maximilians-University of Munich, 82152 Martinsried, Germany; 3 Max Planck Institute for Terrestrial Microbiology, 35043 Marburg, Germany; 4 Institute for General Microbiology, Christian-Albrechts-University, Kiel, Germany; 5 Microverse Cluster, Friedrich Schiller University Jena, 07743 Jena, Germany; 6 Department of Physics, Philipps-Universität Marburg, 35032 Marburg, Germany

**Keywords:** Bacterial genetics, Biofilms

## Abstract

*Vibrio cholerae*, the cause of cholera disease, exhibits a characteristic curved rod morphology, which promotes infectivity and motility in dense hydrogels. Periplasmic protein CrvA determines cell curvature in *V. cholerae*, yet the regulatory factors controlling CrvA are unknown. Here, we discover the VadR small RNA (sRNA) as a post-transcriptional inhibitor of the *crvA* mRNA. Mutation of *vadR* increases cell curvature, whereas overexpression has the inverse effect. We show that *vadR* transcription is activated by the VxrAB two-component system and triggered by cell-wall-targeting antibiotics. *V. cholerae* cells failing to repress *crvA* by VadR display decreased survival upon challenge with penicillin G indicating that cell shape maintenance by the sRNA is critical for antibiotic resistance. VadR also blocks the expression of various key biofilm genes and thereby inhibits biofilm formation in *V. cholerae*. Thus, VadR is an important regulator for synchronizing peptidoglycan integrity, cell shape, and biofilm formation in *V. cholerae*.

## Abstract (web summary)

Small regulatory RNAs (sRNAs) in *Vibrio cholerae* have been shown to modulate several biological processess including virulence, biofilm formation, quorum sensing, colony morphology and stress resistance. Here, the authors show that VadR sRNA acts as a posttranscriptional inhibitor of the *crvA* mRNA and that mutation of *vadR* increases cell curvature, whereas overexpression has the inverse effect.

## Introduction

Bacterial cell shape is highly diverse and tightly conserved at the species level. Certain cell morphologies have been associated with distinct physiological functions such as optimized nutrient uptake, efficient surface adherence, and increased evasion from protist grazing^1^. Cell shape is determined by the geometry of the cell-wall, which can be affected by filamentous proteins that change or interfere with peptidoglycan insertion^2–4^. For example, the cytoskeleton-like filament, crescentin (CreS), controls cell curvature in the model bacterium *Caulobacter crescentus*^5^. In *Vibrio cholerae*, CrvA protein polymerizes in the periplasmic space to promote cell bending^6,7^. *V. cholerae* cells lacking the *crvA* gene display attenuated colonization in animal infection models and it has been reported that cell curvature of *V. cholerae* increases in a cell-density dependent manner^6^. These findings indicate that CrvA levels are continuously adjusted during growth, however, the necessary regulatory factors are currently unknown.

Recently, post-transcriptional control by small regulatory RNAs (sRNAs) in *V. cholerae* was shown to be key for modulating spatiotemporal processes such as virulence, biofilm formation, secondary messenger production, and stress resistance^8–11^. The largest class of sRNAs associates with the RNA chaperone Hfq and typically regulates the expression of target mRNAs by base-pairing via short stretches of imperfect complementarity^12,13^. The network regulated by a single sRNA frequently involves dozens of targets and therefore sRNAs can rival transcription factors with respect to their regulatory scope and biological importance^14^. For example sRNAs are crucial for iron, membrane, and sugar homeostasis, as well as motility, biofilm formation, and virulence^15,16^, however, no sRNA has been yet reported to control cell shape.

Here, we employed the curved rod-shaped bacterium *V. cholerae* as a model system to study the impact of sRNAs on cell curvature. We discovered that production of the VadR (VxrB activated small RNA, see below) sRNA efficiently reduced cell curvature in *V. cholerae* by inhibiting the expression of the *crvAB* mRNA. VadR expression is controlled by the VxrAB two-component system (a.k.a. WigKR^17,18^) and is activated by ß-lactam antibiotics. *V. cholerae* mutants deleted for *vadR* display increased sensitivity towards penicillin and we pinpoint this phenotype to VadR-mediated repression of *crvAB*. In addition, VadR controls several key genes required for biofilm assembly, including the mRNA encoding the RbmA matrix protein^19^. Thus, VadR also inhibits biofilm formation in *V. cholerae*. Our results reveal how a non-coding RNA regulates a cytoskeleton-like filament in bacteria, and establish a link between cell shape, biofilm formation, and antibiotic resistance in *V. cholerae*.

## Results

### VadR is an Hfq-dependent sRNA affecting cell curvature in *V. cholerae*

**Fig. 1:**
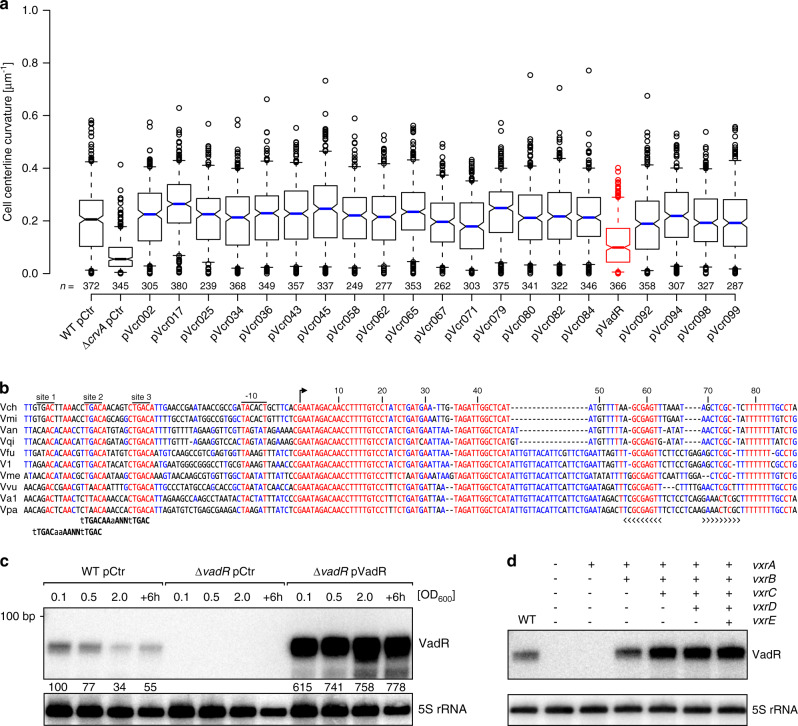
The VadR sRNA alters *V. cholerae* cell shape and is activated by VxrB. **a** Centerline curvature of *V. cholerae* cells expressing the indicated sRNAs (*x*-axis). The blue, black and red lines indicate the median, boxes represent 25th and 75th percentiles, whiskers represent 5th and 95th percentiles and notches indicate 95% confidence intervals for each median. *n* of each set is listed above the *x*-axis over three independent experiments. **b** Alignment of *vadR* and its promoter sequence from various *Vibrio* species (see “Methods” section “Sequence alignment” for details). The -10 box, TSS (arrow) and the Rho-independent terminator (brackets) are indicated. Putative VxrB binding sites and binding motifs (bold) are illustrated. **c** VadR expression throughout bacterial growth was monitored on Northern blots. *V. cholerae* wild-type or *vadR* mutant cells carrying either a control plasmid (pCtr) or a constitutive *vadR* overexpression plasmid (pVadR) were tested. A quantification of VadR expression relative to the wild-type (at OD_600_ of 0.1) is provided. **d** *V. cholerae* Δ*vxrABCDE* cells were complemented with various cistrons of the *vxrABCDE* operon and tested for VadR expression on Northern blots. Expression of the *vxrABCDE* fragments was driven by the inducible pBAD promoter (0.02 % l-arabinose final conc.) and exponentially growing cells were harvested (OD_600_ of 0.2). A *V. cholerae* wild-type strain harboring an empty vector served as control. The experiment was performed with three independent biological replicates (*n* = 3). Source data underlying panels **a**, **c**, and **d** are provided as a Source Data file.

To identify sRNAs regulating cell curvature in *V. cholerae*, we performed a microscopy-based forward genetic screen. We selected 21 uncharacterized sRNAs candidates from a pool of recently identified Hfq-dependent sRNAs^20^ and cloned their respective genes onto multicopy plasmids. We transferred these plasmids into *V. cholerae* and assayed the resulting strains for centerline curvature using phase contrast microscopy. In line with a previous report^6^, we found that curvature decreased ~3-fold in *crvA* deficient cells, when compared to wild-type *V. cholerae* (Fig. 1a). Overexpression of 20 sRNAs did not render curvature significantly, however, cells overexpressing one sRNA, which we term VadR (a.k.a. Vcr090^20^, see below), displayed ~2-fold reduced curvature (Fig. 1a).

The *vadR* gene is located on the plus strand of the smaller *V. cholerae* chromosome between the *vca0002* and *vca0003* genes^20^. The sRNA is present in numerous other *Vibrios* and carries a highly conserved 5′ end (Fig. 1b) frequently involved in RNA duplex formation with *trans*-encoded target mRNAs^8,21^. Structure probing experiments confirmed that this region is unstructured and therefore available for base-pairing with other transcripts (Supplementary Fig. 1a, b). Northern blot analysis revealed that VadR accumulates as a ~85 nt transcript and is most highly expressed at low cell densities (Fig. 1c). Stability of VadR was ~3 min in *V. cholerae* wild-type cells and ~4-fold reduced in cells lacking the *hfq* gene (Supplementary Fig. 1c). Together, we conclude that VadR is a Hfq-dependent sRNA that is likely to act by base-pairing with other transcripts.

### The VxrAB system activates VadR expression

Alignment of *vadR* promoter sequences revealed three conserved elements upstream the −10 box (Fig. 1b). While we were unable to directly assign a transcriptional regulator to these elements, we discovered that a *vadR* transcriptional reporter was ~150-fold more active in *V. cholerae* when compared to *Escherichia coli* (Supplementary Fig. 1d). These results suggested that *vadR* expression depended on a *V. cholerae*-specific factor, which allowed us to perform another genetic screen. Here, we employed a plasmid library expressing ~2.5 kb *V. cholerae* genomic fragments, which we co-transformed with a P*vadR*::*lacZ* transcriptional reporter into *E. coli*. We assayed ~23,000 colonies for ß-galactosidase activity on plates containing X-gal and isolated seven blue colonies. Sequence analysis of the respective plasmids revealed that all mapped to the *vxrABCDE* (*vca0565-0569*) locus; five plasmids contained sequences of *vxrAB* and two plasmids contained sequences of *vxrABCDE* (Supplementary Fig. 1e). To corroborate these results, we monitored *vadR* production in wild-type and Δ*vxrABCDE V. cholerae* by means of (i) promoter activity measurements and (ii) Northern blot analysis. Indeed, promoter activity was ~50-fold reduced in the *vxrABCDE* mutant (Supplementary Fig. 1f) and VadR was no longer detectable on Northern blots (Fig. 1d). Successive complementation of the *vxrABCDE* genes from a plasmid revealed that *vxrAB* (encoding the histidine kinase and response regulator of the two-component system, respectively) restored VadR expression, while *vxrCDE* were dispensable for regulation (Fig. 1d). Finally, to pinpoint direct regulation of *vadR* by VxrB, we reanalyzed previously reported ChIP-Seq data^22^ for binding of VxrB at the *vadR* promoter. Indeed, we discovered a pronounced, VxrB-specific peak upstream of the *vadR* gene (Supplementary Fig. 1g). These analyses also revealed a putative VxrB binding motif (TTGACAAAA-N2-TTGAC), which matched the three conserved sequence elements in the *vadR* promoter (Fig. 1b). Deletion of each of these sites efficiently reduced *vadR* promoter activity with sites 2 and 3 being most critical for transcription activation (Supplementary Fig. 1h). We also tested the effect of deleting the spacer sequence between sites 1 and 2, as well as between sites 2 and 3 on the *vadR* promoter. In both cases, we observed a strong drop in activity (Supplementary Fig. 1h). Together, we conclude that VadR is a VxrAB-activated sRNA that might be involved in regulation of cell shape in *V. cholerae*.

### VadR regulates multiple genes involved in biofilm formation

**Fig. 2:**
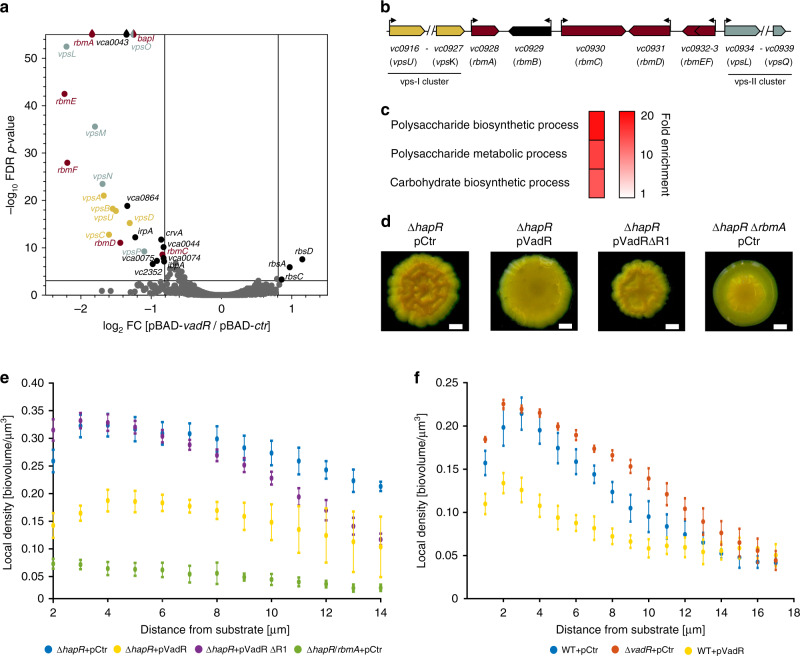
Target spectrum of VadR and its role in biofilm formation. **a** Volcano plot analysis showing differentially regulated genes after pulse induction of VadR. Genes with absolute fold changes ≥1.75 and an FDR (false discovery rate) corrected *p*-value of ≤0.001 were considered significantly expressed and are indicated (FC, fold change). **b** Genomic context of the major biofilm cluster in *V. cholerae*. **c** Gene enrichment analysis of the differentially expressed genes shown in (**a**) using gene ontology analysis^59^. **d** Colony biofilm images of *V. cholerae* Δ*hapR* and Δ*hapR*/Δ*rbmA* cells carrying the indicated plasmids. Each strain was spotted on LB agar plates and incubated for 48 h at room temperature before imaging. Representative images of two independent experiments are shown. Scale bars = 0.5 mm. **e** Confocal spinning disk microscopy was used to monitor biofilm density of *V. cholerae* Δ*hapR* cells carrying the indicated plasmids, as well as Δ*hapR*/Δ*rbmA* mutant harboring a control plasmid. Local cell density as a function of distance from the substratum was plotted for each of the indicated strains from three biologically independent experiments as mean ± SD using the BiofilmQ software^55^. **f** Experiments analogous to **e**, however, *V. cholerae* wild-type and Δ*vadR* cells (carrying the indicated plasmids) were monitored for biofilm density from three biologically independent experiments as mean ± SD. Source data underlying panels **e** and **f** are provided as a Source Data file.

To explore the molecular mechanism of VadR-mediated inhibition of cell bending, we next aimed to identify base-pairing partners of VadR in vivo. We used RNA-seq analyses to assess changes in global transcriptome levels following transient (15 min) overexpression of *vadR* in a Δ*vadR V. cholerae* strain. In total, 28 mRNAs, including *crvA*, displayed significant changes following VadR expression (Fig. 2a and Supplementary Table 1). We validated regulation of all targets, except *ibpA*, using quantitative real-time PCR (by testing all monocistronic genes and the first gene of all regulated operons; Supplementary Fig. 2a). The majority of repressed targets (15) corresponded to a single biofilm gene cluster (*vc0916*-*vc0939*) required for the production of the VPS biofilm exopolysaccharide, as well as genes producing the auxiliary biofilm components, RbmA-F^23^ (Fig. 2b).

Gene ontology (GO) analyses revealed a significant overrepresentation of GO terms associated with polysaccharide synthesis in the downregulated targets (Fig. 2c). Indeed, using the wrinkly colony morphology phenotype of *V. cholerae hapR* deficient cells as a read-out for biofilm formation^20^, we discovered that VadR overexpression resulted in strongly decreased biofilm formation (Fig. 2d). This phenotype was further corroborated by quantitative measurements of biofilm formation of *V. cholerae* Δ*hapR* cells using microfluidic flow chambers and confocal microscopy. Analysis of the respective microscopic images revealed that VadR overexpression resulted in reduced biofilm density (Fig. 2e), and we were able to confirm this phenotype in biofilms formed by *V. cholerae* wild-type cells (Fig. 2f). Reduced biofilm density was previously reported for *V. cholerae* cells lacking the RbmA biofilm matrix protein^19,24,25^ and in accordance with these results and our transcriptomic experiments (Fig. 2a and Supplementary Fig. 2a), we discovered that VadR overexpression led to a ~5-fold decrease in RbmA levels, while *vadR* deletion mildly increased the production of the protein (Supplementary Fig. 2b). Similarly, we observed that *V. cholerae* Δ*vadR* mutants displayed slightly increased biofilm densities when compared to wild-type cells. These results show that in addition to controlling cell shape, VadR also modulates biofilm formation in *V. cholerae*.

### VadR acts post-transcriptionally to repress target gene expression

**Fig. 3:**
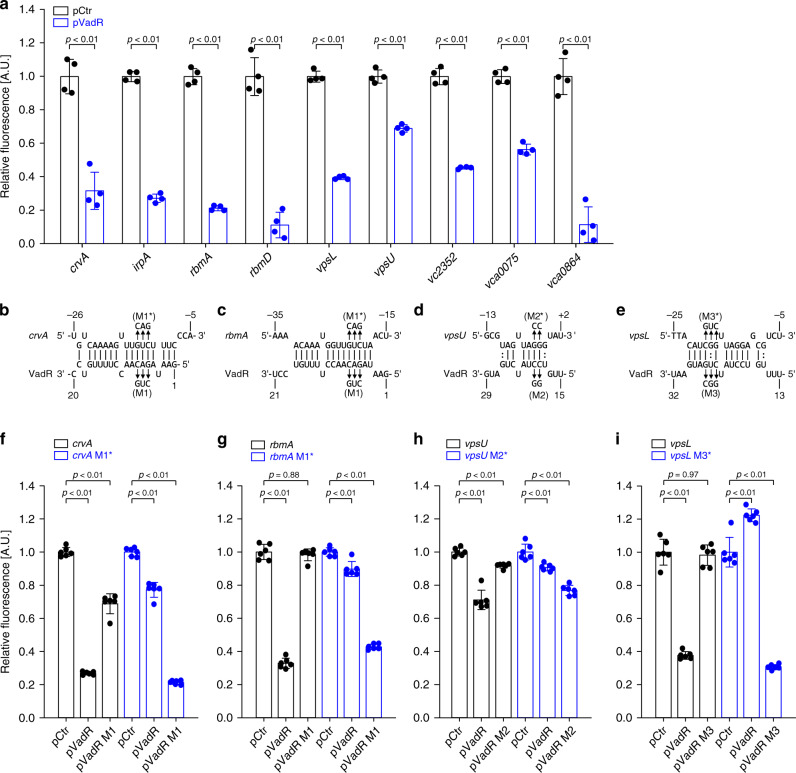
VadR is a direct inhibitor of *crvA* and key biofilm transcripts. **a** Genes post-transcriptionally regulated by VadR. Fluorescence intensities of *E. coli* strains carrying the gene-specific reporters and the control plasmid (pCtr) were set to 1. Bars show mean of biologically independent replicates ± SD, *n* = 4. Statistical significance was determined using one-way ANOVA and post-hoc Sidak tests **b**–**e** Prediction of RNA duplex formation between selected mRNAs and VadR. Numbers indicate the distances from the TSS for VadR and the start codons of the target mRNA sequences, respectively. Arrows indicate the mutations tested in **f**–**i**. **f**–**i** Validation of the predicted mRNA-sRNA duplexes shown in **b**–**e** using compensatory base-pair mutations. Fluorescence levels of *E. coli* strains harboring an empty vector control (pCtr) were set to 1. Bars show mean of biologically independent replicates ± SD, *n* = 6. Statistical significance was determined using one-way ANOVA and post-hoc Sidak tests. Source data underlying panels **a** and **f**–**i** are provided as a Source Data file.

To investigate the molecular underpinnings of VadR-mediated gene control in *V. cholerae*, we cloned the 5′ UTR (untranslated region) and the TIR (translation initiation region) of the 14 potential VadR targets into a GFP-based reporter plasmid designed to score post-transcriptional control^26^. Co-transformation of these plasmids with a VadR overexpression vector or a control plasmid in *E. coli* confirmed post-transcriptional repression of nine targets (*crvA*, *irpA*, *rbmA*, *rbmD*, *vpsL*, *vpsU*, *vc2352*, *vca0075*, and *vca0864*), while we were unable to validate direct regulation of *bapI*, *rbmC*, *rbmF*, *rbsD*, and *vca0043* (Fig. 3a and Supplementary Fig. 3a). We currently do not know why we were unable to confirm regulation of these genes in *E. coli*, however, it is possible that VadR forms RNA duplexes with the deep coding sequence of their respective mRNAs, which would not be captured in our GFP reporters. Using the RNA hybrid algorithm^27^, we next predicted RNA duplex formations of VadR with *crvA*, *rbmA*, *vpsU*, and *vpsL* (Fig. 3b–e). In all four cases, the potential pairing involved the target’s TIR and sequence elements located in the first 30 nucleotides of VadR. Using compensatory base-pair exchange experiments (creating mutants M1, M2, and M3 in *vadR*, see Supplementary Figs. 1a and 3b), we validated binding at the predicted positions (Fig. 3f–i). To bolster these results at the phenotypic level, we also tested the effect of a VadR variant (VadRΔR1, see Supplementary Figs. 1a and 3b) unable to repress three of the four target genes on biofilm formation of *hapR*-deficient *V. cholerae*. In contrast to wild-type VadR, VadRΔR1 did not affect biofilm architecture (Fig. 2d, e), suggesting that VadR acts post-transcriptionally to modulate biofilm formation in *V. cholerae*.

### VadR controls cell shape by inhibiting CrvA production

**Fig. 4:**
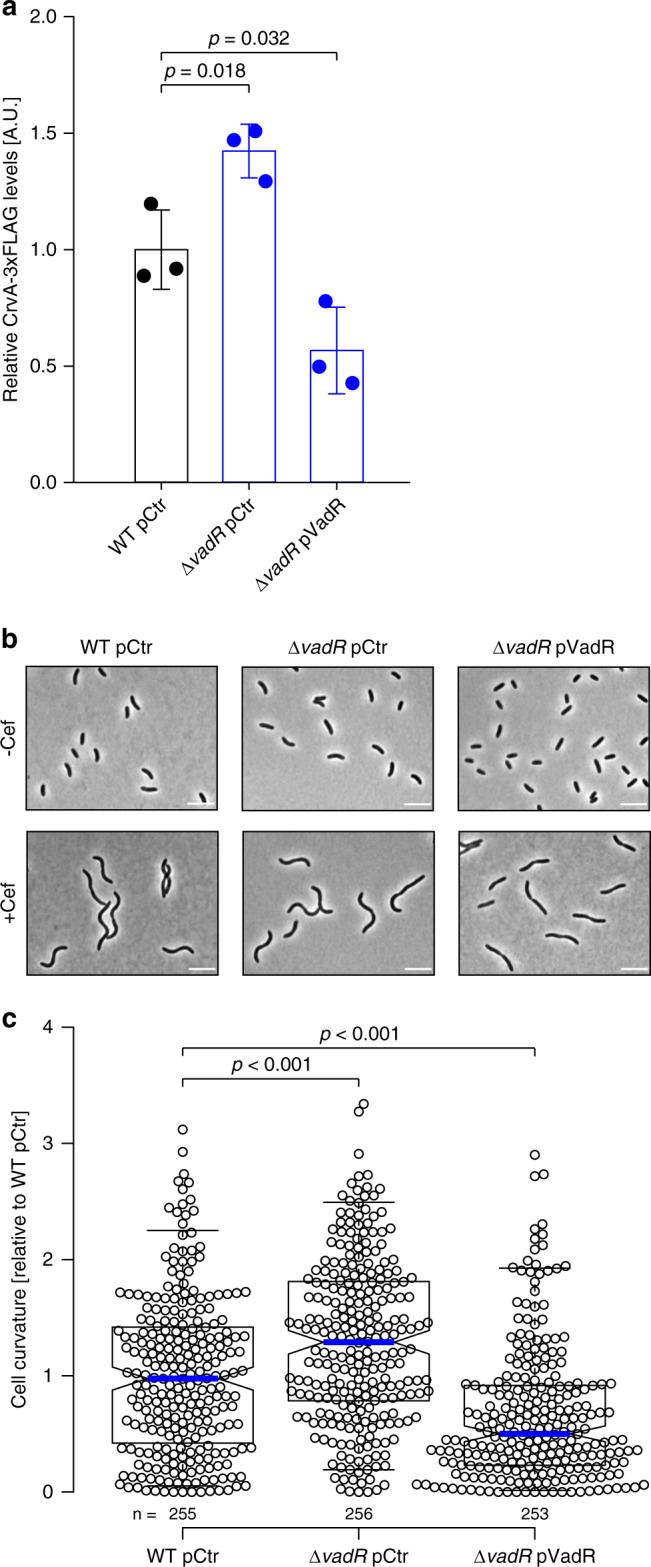
VadR modulates *V. cholerae* curvature by repressing CrvA. **a** Quantification of CrvA-3xFLAG protein levels in *V. cholerae* wild-type and *vadR-*deficient cells (carrying the indicated plasmids). Total protein samples of the indicated strains were harvested (OD_600_ of 0.5) and tested by Western blot analysis. CrvA-3xFLAG protein levels detected in the wild-type cells were set to 1. Bars show mean of biological replicates ± SD, *n* = 3. Statistical significance was determined using one-way ANOVA and post-hoc Holm-Sidak test. Samples of the three biologically independent replicates were processed in parallel. **b** Microscopy of cells used in (**a** –Cef). A second set of cells was treated with cefalexin for 1 h (+Cef) after reaching an OD_600_ of 0.5. Shown are representative fields of vision (biological replicates *n* = 3). Scale bars = 5 µm. **c** Analysis of cell centerline curvature in –Cef samples of **b**. The curvature mean of wild-type cells was set to 1. A blue line indicates the median, boxes represent 25th and 75th percentiles, whiskers represent 5th and 95th percentiles and notches indicate 95% confidence intervals for each median. *n* of each set is listed above the *x*-axis over three independent experiments. Statistical significance was determined using one-sided Kruskal-Wallis test and post-hoc Dunn’s test. Source data underlying panels **a** and **c** are provided as a Source Data file.

Our previous data revealed VadR as a direct repressor of the *crvA* mRNA (Fig. 3b, f) and hence we next aimed to study the role of VadR in cell curvature in *V. cholerae*. Western blot analysis showed that CrvA levels were ~1.5-fold elevated in Δ*vadR* cells, whereas VadR overexpression led to a ~2-fold reduction in CrvA production (Fig. 4a). We correlated these results with microscopic curvature analyses of single cells and discovered that *vadR*-deficient mutants displayed increased curvature, whereas plasmid-borne VadR production had the reverse effect (Fig. 4b, c top). This effect was further amplified when cells were treated with sub-inhibitory concentrations of cefalexin forcing filamentation in *V. cholerae* (Fig. 4b, bottom). Importantly, neither *vadR* deletion, nor its overexpression affected cell length or volume of *V. cholerae* (Supplementary Fig. 4a, b), indicating that VadR specifically modulates cell curvature by inhibiting *crvA* expression.

### VadR-mediated repression of *crvA* affects antibiotic resistance

**Fig. 5:**
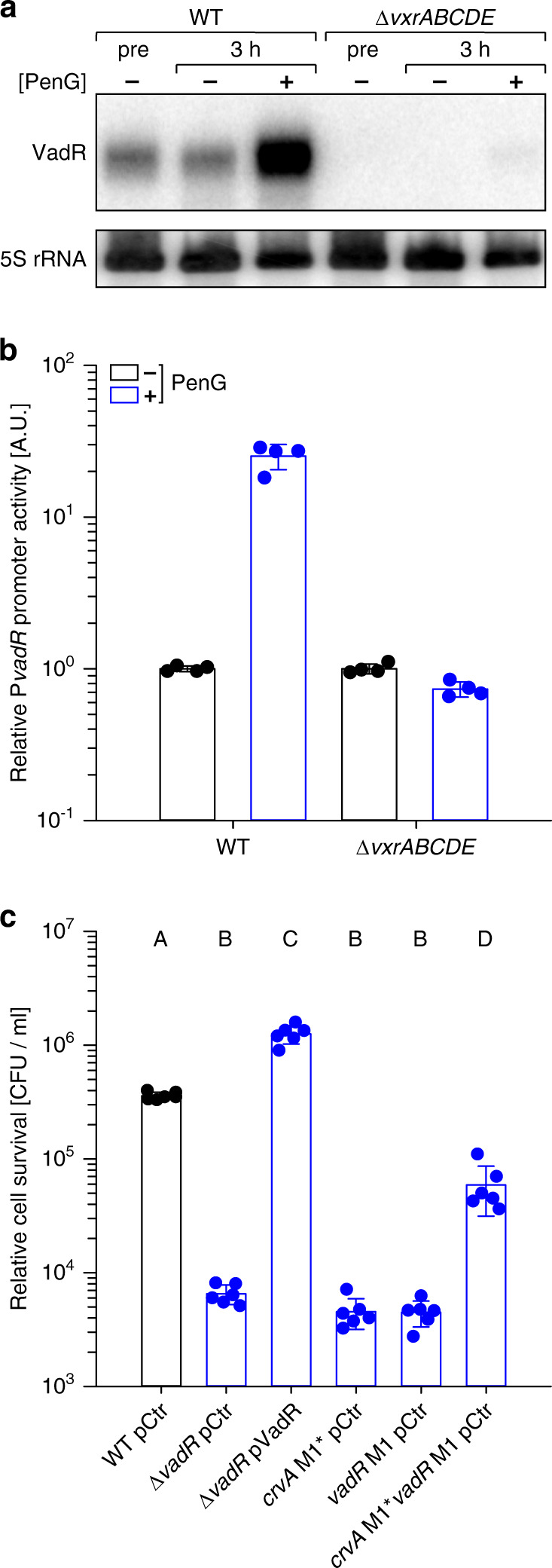
VadR mediates ß-lactam resistance through repression of *crvA*. **a** *V. cholerae* wild-type and *vxrABCDE* mutant strains were grown to OD = 0.2 (pre) and split into two sets. One set was treated with penicillin G, while the other set received a mock treatment. After 3 h, RNA was isolated and VadR expression was monitored by Northern analysis. **b** VadR promoter activity was tested under the same conditions as in **a** using a fluorescent transcriptional reporter. Promoter activities of mock-treated strains were set to 1. Bars represent mean of biologically independent replicates ± SD, *n* = 4. **c** The indicated *V. cholerae* strains (*x*-axis) were grown to OD_600_ = 0.4 and treated with penicillin G for 3 h. Survival after treatment was determined by counting colony forming units (CFUs). Bars represent mean of biologically independent replicates ± SD, *n* = 6. Statistical significance was determined using one-way ANOVA and post-hoc Holm-Sidak test. Significantly different groups (*p* < 0.01) are labeled with corresponding letters. Source data underlying panels **a**–**c** are provided as a Source Data file.

CrvA regulates cell curvature by spatially modulating peptidoglycan insertion in *V. cholerae*^6^ and the VxrAB regulon is induced by peptidoglycan-targeting antibiotics such as penicillin G^18^. Consequently, we tested the effect of penicillin G on VadR expression. Indeed, Northern blot analysis showed ~7-fold increased VadR levels in *V. cholerae* wild-type cells following treatment with penicillin G (Fig. 5a) and we observed ~25-fold induction when we tested *vadR* promoter activity using a transcriptional reporter (Fig. 5b). In both cases, penicillin G-dependent activation of *vadR* was abrogated in the Δ*vxrABCDE* strain (Fig. 5a, b). Expression of *vadR* was also activated by the MreB-targeting antibiotic A22^28^, albeit to a lower extent when compared to penicillin G (Supplementary Fig. 5a).

Based on these results, we speculated that modulation of cell shape by VrxAB and VadR might affect the sensitivity of *V. cholerae* towards cell-wall damaging antibiotics. Following this hypothesis, we first determined the effect of penicillin G treatment on VadR-mediated CrvA repression. Indeed, following treatment of *V. cholerae* with penicillin G, we discovered ~3.5-fold higher CrvA levels in Δ*vadR* cells when compared to wild-type *V. cholerae* (Supplementary Fig. 5b). In addition, we also discovered reduced penicillin G survival rates for *vadR*-deficient *V. cholerae* cells and we were able to complement this phenotype using plasmid-borne VadR production (Fig. 5c). To pinpoint this effect to VadR-mediated repression of *crvA* in the presence of penicillin G, we introduced mutation M1* (Fig. 3b) at the chromosomal *crvA* locus. This mutation does not abrogate *crvA* expression (Supplementary Fig. 5c), but renders the transcript immune towards post-transcriptional repression by VadR. This strain phenocopied the effect of a *vadR* mutant and we obtained almost identical results when we introduced the corresponding mutation (M1, Fig. 3b and Supplementary Fig. 1a) at the chromosomal *vadR* gene (Fig. 5c). Combination of the two mutant alleles resulted in a partial restoration of penicillin G resistance (Fig. 5c), indicating that VadR might be required to mitigate the detrimental effect of CrvA under antibiotic pressure. Notably, neither mutation nor overexpression of *vadR* affected survival of *V. cholerae* under standard growth conditions (Supplementary Fig. 5d).

### VadR is expressed during early biofilm formation

**Fig. 6:**
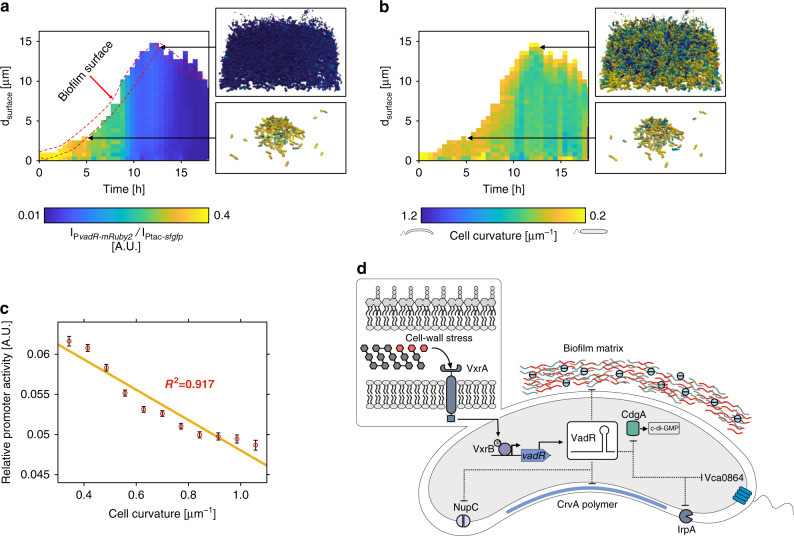
VadR controls cell curvature during biofilm development. **a** Relative activity of the *vadR* promoter during biofilm growth of wild-type *V. cholerae* cells. In each cell the fluorescence of mRuby2, expressed from the *vadR* promoter was normalized by the signal of the constitutive Ptac-promoter-driven sfGFP-fluorescence signal. Heatmap shows *vadR* promoter activity at both spatial (distance from surface of biofilm representing height of the biofilm) and temporal (time of biofilm growth) resolution. Subset of images show the cells from two time points and separate locations of the biofilm. These cells were rendered by ParaView^56^ after final segmentation and analysis using BiofilmQ^55^. The color of each cell represents the activity of the *vadR* promoter. **b** Spatio-temporal heatmap showing cell curvature of each cell for *V. cholerae* biofilms. Cell curvature of individual cells was calculated using BiofilmQ^55^. To calculate the cell curvature of each cell inside the biofilms, similar positions of the biofilm as in **a** were selected for rendering. In these subset of images, the color represents the cell curvature of each cell. **c** A correlation graph was plotted for *vadR* promoter activity as function of cell curvature. Calculation of *vadR* promoter activity and cell curvature was done for *V. cholerae* wild-type biofilms grown in flow chambers. Each point represents the mean ± SEM of >1000 cells for given time point in a biofilm. **d** Model showing the regulatory functions of the VadR sRNA in *V. cholerae*. Expression of *vadR* sRNA is controlled by the VxrAB two-component system. The sRNA regulates multiple biological processes, including cell shape and biofilm formation. Source data underlying panels **a**–**c** are provided as a Source Data file.

To connect the roles of VadR in cell curvature regulation and biofilm formation, we monitored VadR expression (using a P*vadR*::mRuby2 transcriptional reporter) in growing biofilms of *V. cholerae* wild-type cells employing single-cell confocal microscopy analysis^29^. When normalized for sfGFP production driven from the constitutive *P*_tac_ promoter, we discovered that the *vadR* promoter is most active during the initial phases of biofilm formation, while expression is switched off in mature biofilms (Fig. 6a). In parallel, we also determined cell curvature of individual cells during biofilm development (Fig. 6b). Comparison of the two datasets showed that VadR expression and cell curvature are negatively correlated (Fig. 6c), suggesting that VadR expression results in straighter cells during early phases of biofilm development, whereas mature biofilms are more likely to contain a higher proportion of curved cells.

## Discussion

There is an ever growing list of microbial functions and phenotypes involving RNA-based gene regulation. Here, we have identified the Hfq-dependent VadR sRNA as regulator of cell shape and biofilm formation in *V. cholerae*. While sRNA-mediated control of microbial cell shape was previously unknown, other sRNAs have been reported to modulate biofilm formation in *V. cholerae* and other species^30^. For example, we have previously shown that the VqmR sRNA also inhibits biofilm formation in *V. cholerae*^20,31^, however, instead of base-pairing with the mRNAs of the multiple biofilm components (as shown here for VadR), VqmR inhibits translation of *vpsT*, encoding a transcriptional activator of biofilm genes in *V. cholerae*^32^. Thus, although VadR and VqmR both inhibit biofilm formation, VqmR acts at a higher level of the biofilm pathway. This difference in regulatory hierarchy could also point to a conceptual difference in the underlying regulatory principle of how these two sRNAs fulfill their function in the cell. Repression of *vpsT* translation by VqmR will become effective when VpsT protein levels decreased sufficiently to affect the transcription of biofilm-related genes and thus might be considered to be part of larger regulatory process that mediates the switch from low-cell to high-cell density behaviors in *V. cholerae*^9^. In contrast, inhibition of multiple biofilm genes by VadR should produce a more immediate response to adjust the production of biofilm factors to the status of VxrAB system.

Interestingly, the VxrAB system has previously been shown to induce biofilm formation in *V. cholerae*^33^, which at first glance might seem counterintuitive given that VadR is also induced by VxrAB and inhibits biofilm formation. Nevertheless, bacterial sRNAs are known to be frequently part of mixed regulatory networks involving transcriptional regulators as well as noncoding RNAs^34^. It is currently unclear at which level VxrAB function to activate biofilm formation, however, overexpression of VxrB induces the *vpsL* promoter^33^ and the *vpsL* mRNA is repressed by VadR (Fig. 3i). It is therefore possible that VxrB, VadR, and *vpsL* form a type 1 incoherent feed-forward loop (I1-FFL), in which the top activator regulates both a gene and a repressor of the gene. I1-FFL have been reported to create pulse-like gene expression dynamics and to accelerate the response time of a system^35^. In addition, I1-FFL can reduce gene expression noise^36^, which is particularly relevant when the activity of the upstream transcriptional activator (here VxrB) fluctuates. The exact signal triggering the VxrAB system is currently unknown, however, in the closely related species *Vibrio parahaemolyticus*, the VxrAB system (a.k.a. VbrKR) has been reported to respond to ß-lactam antibiotics via direct interaction with the histidine kinase, VbrK^37^. Our results support activation of the system by ß-lactam antibiotics, i.e., penicillin G (Fig. 5a, b), however, since we also discovered *vadR* activation in the presence of A22 (Supplementary Fig. 5a), it is likely that additional cues also trigger the system.

In fact, VadR expression is readily detectable under standard growth conditions (Fig. 1c) suggesting a regulatory role for the system under non-stress conditions. Here, VadR might take the role of adjusting cell growth with the production of CrvA and biofilm-forming factors (Fig. 6d). CrvA is an abundant periplasmic protein^6^ and biofilm components require transport across two membranes to reach their final destination^23^. Uncoordinated export of proteins and polysaccharides can clog the cellular transport machineries and compromise the permeability barrier or structural integrity of the cell^38,39^. It is therefore vital for the cell to synchronize these functions with cell growth and sRNAs have previously been implicated in this process^40^. For example, sRNAs activated by the alternative sigma-factor E promote envelope homeostasis by tuning the levels of newly synthesized outer membrane proteins in response to misfolded proteins in the periplasm^8,21,41,42^. VadR could take an analogous position in the VxrAB stress response system and given the relatively short half-life of VadR (~3 min, Supplementary Fig. 1c), sRNA-based regulation might provide regulatory dynamics that are superior over canonical protein-based regulation^14^.

How CrvA affects peptidoglycan remodeling in *V. cholerae* is currently not fully understood. Previous reports indicated that filament-like proteins such as CrvA and CreS render the activity of enzymes involved in cell-wall synthesis and thereby reduce the rate of peptidoglycan insertion at one site of the cell^6,43^. This process results in asymmetric growth and cell curvature. Indeed, when comparing incorporation of HADA (HCC-amino-d-alanine^44^) into peptidoglycan of *V. cholerae* wild-type and VadR-overexpressing cells, we observed that HADA insertion was biased towards the outer surface of wild-type cells, while VadR-overexpression restored this effect (Supplementary Fig. 6a). Given that CrvA does not affect the overall peptidoglycan composition in *V. cholerae*^6^, it might be possible that the relative increase in peptidoglycan synthesis at the outer surface facilitates the activity of ß-lactam antibiotics at this side and thus stimulates cell wall lysis. This idea is supported by our finding that penicillin-treated *V. cholerae* wild-type cells are more prone to form spheroplasts, when compared to VadR-overexpressing cells (Supplementary Fig. 6b). Taken together, VadR-mediated repression of *crvA* mRNA could help to mitigate curvature-associated antibiotic sensitivity by reducing the de novo production of CrvA protein under stress conditions.

While adjusting cell curvature in response to cell growth and antibiotic stress could be an important feature of VadR-mediated gene regulation in *V. cholerae*, it is relevant to note that not all *Vibrios* encode the *crvA* gene (e.g., *V. parahaemolyticus*) and thus do not display a curved cell morphology^6^. However, *vadR* and *vxrAB* are highly conserved in these organisms (Fig. 1b and Supplementary Fig. 7) suggesting that VadR’s role goes beyond cell curvature control. The *vpsL and vpsU* genes are highly conserved among the biofilm-associated factors repressed by VadR, while *rbmA* and *rbmD* show only poor conservation (Supplementary Fig. 7). Additional targets of VadR are *vc2352* (encoding a NupC-type nucleoside transporter^45^), *irpA* (encoding an iron-regulated membrane protein carrying a peptidase domain^46^), *vca0864* (encoding a methyl-accepting chemotaxis protein^47^), and *vca0075* (function unknown^48^), all of which display high conservation among the *Vibrios* (Figs. 3a, 6d and Supplementary Fig. 7). Interestingly, *vca0075* is co-repressed with *cdgA*^20^, a diguanylate cyclase gene with documented functions in biofilm formation^49^. In addition, Vca0864 has been reported to inhibit chemotaxis towards N-acetylglucosamine, which is a key for peptidoglycan synthesis^50^. We currently do not yet know how these genes fit into the VadR regulon, however, understanding their biological functions might help to obtain a more comprehensive view on the VadR regulon in *V. cholerae* and related *Vibrios*.

## Methods

### Bacterial strains and growth conditions

Bacterial strains used in this study are listed in Supplementary Table 2. Details for strain construction are provided in the Supplementary Material and Methods section. *V. cholerae* and *E. coli* cells were grown under aerobic conditions (200 rpm, 37 °C) in LB (Lennox Broth). Where appropriate, media were supplemented with antibiotics at the following concentrations: 100 µg mL^−1^ ampicillin; 20 µg mL^−1^ chloramphenicol; 50 µg mL^−1^ kanamycin; 50 U mL^−1^ polymyxin B; 5 mg mL^−1^ streptomycin, 5 µg mL^−1^ cefalexin, and 50 µg mL^−1^ penicillin G.

### Plasmids and DNA oligonucleotides

All plasmids and DNA oligonucleotides used in this study are listed in Supplementary Tables 3 and 4, respectively. Cloning details are provided in the Supplementary Material and Methods section.

### RNA isolation and Northern blot analysis

Total RNA was prepared and blotted as described previously^51^. Membranes (GE Healthcare Amersham) were hybridized with [^32^P] labeled DNA oligonucleotides at 42 °C. Signals were visualized using a Typhoon phosphorimager (GE Healthcare) and quantified using Gelquant software (biochemlabsolutions).

### Screen for curvature-regulating sRNAs and microscopy analysis

*V. cholerae* wild-type cells were conjugated with either an empty control plasmid (pCMW-1K) or overexpression plasmids of the following sRNAs: Vcr002, Vcr017, Vcr025, Vcr034, Vcr036, Vcr043, Vcr045, Vcr058, Vcr062, Vcr065, Vcr067, Vcr071, Vcr079, Vcr080, Vcr082, Vcr084, VadR, Vcr092, Vcr094, Vcr098, or Vcr099. For microscopy analyses, the respective *V. cholerae* strains were cultivated in LB to OD_600_ of 0.4. Cells were pelleted, washed in 1× PBS, and resuspended in 2.5% paraformaldehyde in 1× PBS. Phase contrast imaging was performed on a Zeiss Axio Imager M1 microscope equipped with EC Plan Neofluar 100×/ 1.3 Oil Ph3 objective (Zeiss). For further analyses, e.g., measurements of cell center line curvature, cell length and cell area, the FIJI-plugin MicrobeJ was used^52,53^. Colony biofilms were imaged with Stemi 305 stereoscopic microscope (Zeiss) and acquired with Labscope (Zeiss).

### Flow chamber biofilms and confocal imaging

The strains were grown in LB medium supplemented with 50 μg mL^−1^ kanamycin, to mid-exponential growth phase, before introducing into microfluidic flow chambers. Flow chambers were constructed from poly(dimethylsiloxane) bonded to glass coverslips using an oxygen plasma. The microfluidic channels measured 500 μm in width, 100 μm in height and 7 mm in length. After the cultures were introduced into the channels, the channels were incubated at 24 °C for 1 h without any flow, to allow cells to attach to the bottom glass surface of the channels. The flow was then set to 0.3 μL min^−1^ for approximately 18 h before images were acquired. Cells were stained with green fluorescent nucleic acid stain dye, SYTO 9 (Thermo Fisher Scientific), by exchanging the syringes containing LB with SYTO 9 for 30 min. Flow rates were controlled using a high-precision syringe pump (Pico Plus, Harvard Apparatus). To acquire the spatiotemporal information of individual cells in a growing biofilm, time lapse confocal microscopy was performed as described previously^54^. To reduce photobleaching and phototoxicity during time-lapse imaging, a live feedback between image acquisition, image analysis and microscope control was used to automatically detect the biofilm height to avoid imaging of empty space on top of the biofilms. Images were acquired with an Olympus 100× objective with numerical aperture of 1.35, using a Yokogawa spinning disk confocal scanner and laser excitation at 488 nm. Images were acquired at spatial resolution of 63 nm in the *xy*-plane and 400 nm along the *z*-direction. To detect all single cells, measure cell curvature of each cell, and quantify the relative *vadR* promoter-reporter strength from biofilm grown in flow chambers, biofilm images were analysed using the BiofilmQ software^55^. Kymograph heatmaps showing the strength of *vadR* promoter and cell curvature during biofilm growth were generated with BiofilmQ. 3-D cell rendering was done using BiofilmQ-analysed biofilm data using the ParaView software^56^. Biofilm images were prepared with the NIS-Elements AR Analysis software (Nikon) by cropping a fixed *z*-plane with *xy* and *yz* projections.

### RNA-seq analysis

Biological triplicates of *V. cholerae* Δ*vadR* strains harboring pBAD-Ctr or pBAD-*vadR* plasmids were grown to exponential phase (OD_600_ of 0.2) in LB media. sRNA expression was induced by addition of l-arabinose (0.2% final conc.). After 10 min of induction, cells were harvested by addition of 0.2 volumes of stop mix (95% ethanol, 5% (v/v) phenol) and snap-frozen in liquid nitrogen. Total RNA was isolated and digested with Turbo DNase (Thermo Fischer Scientific). Ribosomal RNA was depleted using Ribo-Zero kits (Epicenter) for Gram-negative bacteria, and RNA integrity was confirmed using a Bioanalyzer (Agilent). Directional cDNA libraries were prepared using the NEBNext Ultra II Directional RNA Library Prep Kit for Illumina (NEB, #E7760). The libraries were sequenced using a HiSeq 1500 system in single-read mode for 100 cycles. The read files in FASTQ format were imported into CLC Genomics Workbench v11 (Qiagen) and trimmed for quality and 3′ adapters. Reads were mapped to the *V. cholerae* reference genome (NCBI accession numbers: NC_002505.1 and NC_002506.1) using the “RNA-Seq Analysis” tool with default parameters. Reads mapping to annotated coding sequences were counted, normalized (CPM) and transformed (log_2_). Differential expression between the conditions was tested using the “Empirical Analysis of DGE” command. Genes with a fold change ≥1.75 and an FDR adjusted *p*-value ≤ 0.001 were defined as differentially expressed.

### Fluorescence measurements

Fluorescence assays to measure GFP expression were performed as described previously^26^. *E. coli* strains expressing translational GFP-based reporter fusions were grown for 16 h in LB medium and resuspended in 1× PBS. Fluorescence intensity was quantified using a Spark 10 M plate reader (Tecan). *V. cholerae* and *E. coli* strains carrying mKate2 transcriptional reporters were grown in LB medium, resuspended in 1× PBS, samples were collected at the indicated time points and mKate2 fluorescence was measured using a Spark 10 M plate reader (Tecan). Control samples not expressing fluorescent proteins were used to subtract background fluorescence.

### Western blot analysis

Experiments were performed as previously described^57^. Protein samples were separated using SDS-PAGE and transferred to PVDF membranes for Western blot analysis. 3× FLAG-tagged fusions were detected using anti-FLAG antibody (Sigma, #F1804). RnaPα served as a loading control and was detected using anti-RnaPα antibody (BioLegend, #WP003). Signals were visualized using a Fusion FX EDGE imager (Vilber) and band intensities were quantified using the BIO-1D (Vilber) or Gelquant software (biochemlabsolutions).

### Sequence alignment

VadR and its promoter sequences among various *Vibrio* species were aligned using the MultAlin webtool^58^. Vch: *Vibrio cholerae* (NCBI:txid243277), Vmi: *Vibrio mimicus* (NCBI:txid1267896), Van: *Vibrio anguillarum* (NCBI:txid55601), Vqi: *Vibrio qinghaiensis* (NCBI:txid2025808), Vfu: *Vibrio furnissii* (NCBI:txid29494), Vfl: *Vibrio fluvialis* (NCBI:txid676), Vme: *Vibrio mediterranei* (NCBI:txid689), Vvu: *Vibrio vulnificus* (NCBI:txid672), Val: *Vibrio alginolyticus* (NCBI:txid663), and Vpa: *Vibrio parahaemolyticus* (NCBI:txid670).

### Statistical analyses

Statistical parameters for the respective experiment are indicated in the corresponding figure legends. *n* represents the number of biological replicates. Details for the performed statistical tests are provided in the supporting information. Statistical analyses of CFUs were performed as follows: The data were log_10_-transformed and tested for normality and equal variance using Kolmogorov–Smirnov and Brown–Forsythe tests, respectively. The data were tested for significant differences using one-way ANOVA and posthoc Holm-Sidak tests. Significance levels are reported in the in the supporting information. Statistical analysis was performed using SigmaPlot v14 (Systat) and GraphPad Prism 8 (GraphPad Software). No blinding or randomization was used in the experiments. No estimation of statistical power was used before performing the experiments and no data were excluded from analysis.

### Reporting summary

Further information on research design is available in the Nature Research Reporting Summary linked to this article.

## Supplementary information

Supplementary Information

Reporting Summary

## Data Availability

The raw data of the transcriptome analyses are available at the National Center for Biotechnology Information Gene Expression Omnibus (GEO) under the accession number GSE145764. Source data are provided with this paper.

## Source data

Source Data

## Code availability

The biofilm image analysis software tool BiofilmQ^55^ is available online (https://drescherlab.org/data/biofilmQ/ and 10.1101/735423). HADA-profiles of outer and inner cell surfaces were determined by using a custom method, developed by FIJI. The respective code is part of yet unpublished work and available upon request.
