# Supplementary material for: RNA-mediated control of cell shape modulates antibiotic resistance in Vibrio cholerae
Source: Nat Commun. 2020 Nov 27;11:6067. doi: 10.1038/s41467-020-19890-8 (PMC7695739; doi:10.1038/s41467-020-19890-8)
Supplement: Supplementary file 1 — Supplementary Information [file 41467_2020_19890_MOESM1_ESM.pdf]

RNA-mediated control of cell shape modulates antibiotic resistance in *Vibrio cholerae*

Nikolai Peschek[1,2*], Roman Herzog[1,2*], Praveen K. Singh[3*], Marcel Sprenger[1*], Fabian Meyer[2,4], Kathrin S. Fröhlich[1,2], Luise Schröger[2], Marc Bramkamp[2,4], Knut Drescher[3,5], and Kai Papenfort[1,2,6#]

[1] Friedrich Schiller University, Institute of Microbiology, 07745 Jena, Germany

[2] Faculty of Biology I, Ludwig-Maximilians-University of Munich, 82152 Martinsried, Germany

[3] Max Planck Institute for Terrestrial Microbiology, 35043 Marburg, Germany

[4] Institute for General Microbiology, Christian-Albrechts-University, Kiel, Germany,

[5] Department of Physics, Philipps-Universität Marburg, 35032 Marburg, Germany

[6] Microverse Cluster, Friedrich Schiller University Jena, 07743 Jena, Germany.

* These authors contributed equally

# Corresponding author:
Kai Papenfort
E-mail: kai.papenfort@uni-jena.de
Phone: +49-3641-949311

This supplement contains:

Supplementary Figures 1-7
Supplementary Materials and Methods
Supplementary Tables 1-4
Supplemental References

TABLE OF CONTENTS

Supplementary Fig. 1 Structure and transcriptional control of the VadR sRNA

Supplementary Fig. 2 RNA-seq target validation and VadR-mediated regulation of RbmA

Supplementary Fig. 3 Potential VadR target genes and expression of VadR variants

Supplementary Fig. 4 VadR does not affect the length and area of *V. cholerae* cells

Supplementary Fig. 5 The VadR promoter responds to A22 treatment and *V. cholerae* depends on tight *crvA* regulation to overcome Penicillin G stress

Supplementary Fig. 6 Microscopic analysis of *V. cholerae* cells using HADA labelling.

Supplementary Fig. 7 Conservation of VadR targets genes among different *Vibrio* species.

Supplementary Materials and Methods

Supplementary Table 1 VadR RNA-seq

Supplementary Table 2 Bacterial strains used in this study

Supplementary Table 3 Plasmids used in this study

Supplementary Table 4 DNA oligonucleotides used in this study

Supplementary Figures

Supplementary Figure 1

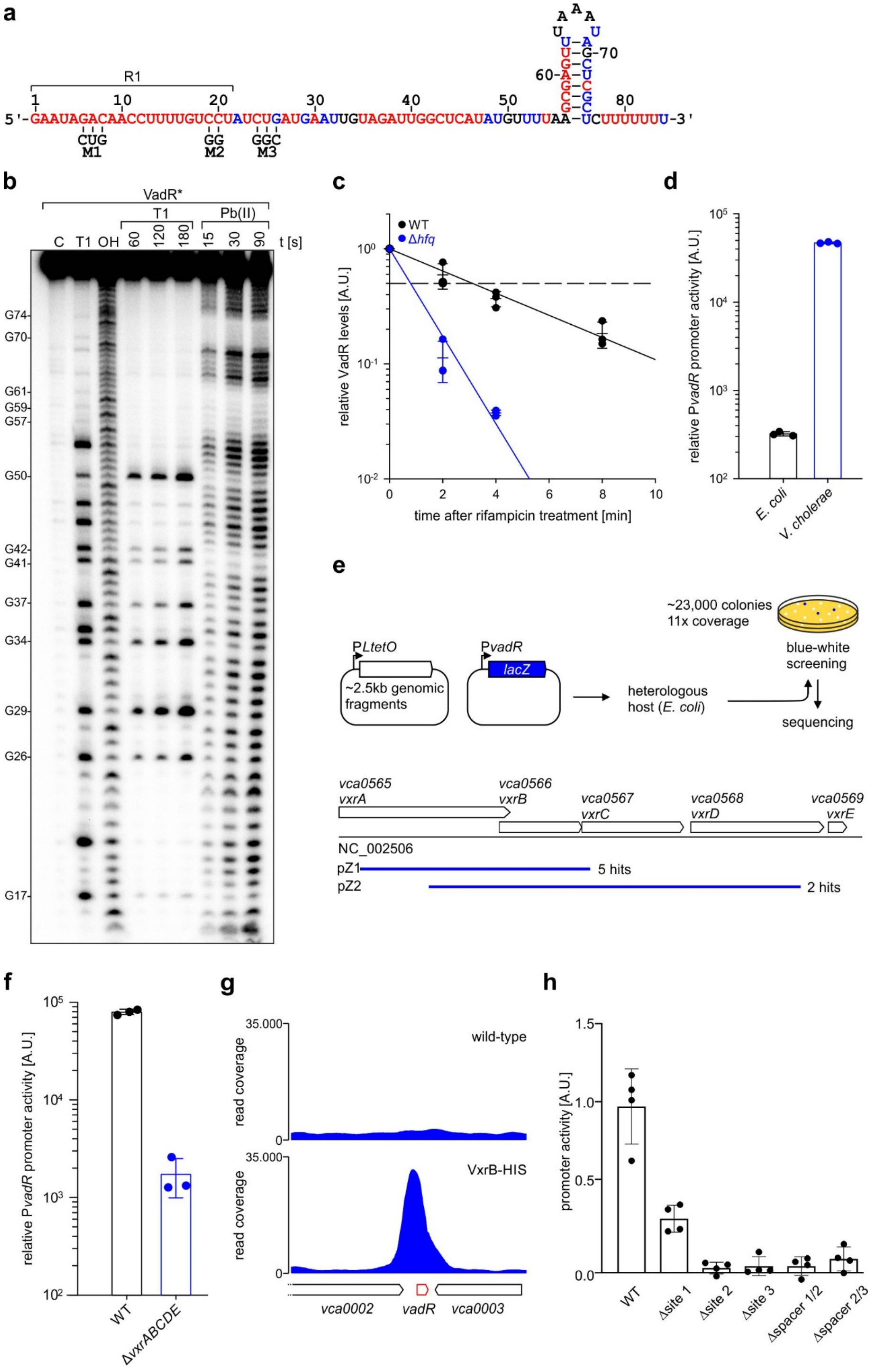

Supplementary Figure 1: Structure and transcriptional control of the VadR sRNA

a The secondary structure of the VadR sRNA predicted by structure probing experiments (b).

b Secondary structure probing of the VadR sRNA. VadR was synthesized *in vitro* and labelled with ^{32}P. Enzymatic treatment was performed using RNase T1, or lead-acetate (Pb(II)). The untreated control is labelled with C, denatured ladders for RNase T1 and alkaline ladder are provided and labelled with T1 and OH, respectively. Guanin residues are labelled on the left side. The experiment was done in two biological replicates.

c Rifampicin treatment to determine half-life of the VadR sRNA, in wild-type or *hfq* mutant strains. The dashes represent the mean of biologically independent replicates ± SD, n = 3.

d VadR promoter activities in *E. coli* and *V. cholerae* cultures grown for 16h in LB were determined using a fluorescent transcriptional reporter. Data are the mean of biologically independent replicates ± SD, n = 3.

e Upper part: Experimental outline to identify transcription factors affecting *vadR* transcription. Lower part: Identified fragments that yielded blue colonies.

f *V. cholerae* wild-type and *vxrABCDE* mutant strains were grown to OD_{600} = 0.5 and VadR promoter activities were measured. Bars show the mean of biologically independent replicates ± SD, n = 3.

g ChIP-seq data from *V. cholerae* wild-type and *vxrB-HIS* strains, which were treated with penicillin G for 3 h[1]. Data was re-analyzed and read coverages for the *vadR* genomic locus were plotted.

h Three putative VxrB binding sites and the spacer between the binding sites 1/2 and 2/3 in the promoter region of *vadR* were deleted. The resulting strains and *V. cholerae* wild-type were cultivated to OD_{600} = 1.0 and assayed for *vadR* promoter activities using a fluorescent transcriptional reporter. Bars represent the mean of biologically independent replicates ± SD, n = 4. The mean of wild-type cells was set to 1. Source data underlying panels **b**, **c**, **d**, **f** and **h** are provided as a Source Data file.

Supplementary Figure 2

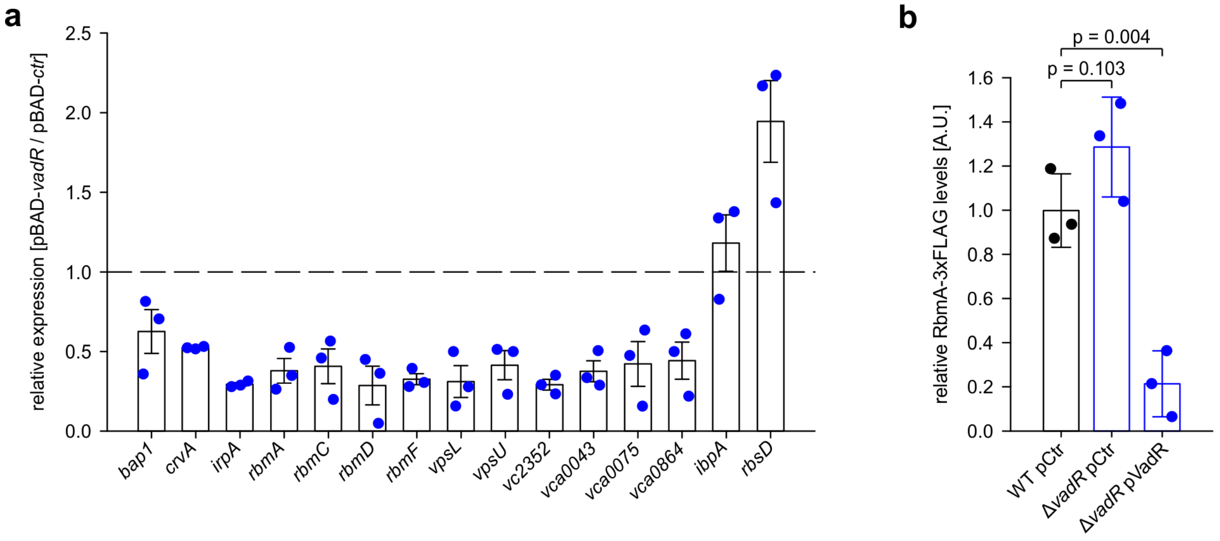

Supplementary Figure 2: RNA-seq target validation and VadR-mediated regulation of RbmA

a qRT-PCR analysis after short period VadR expression. Expression was calculated relative to an empty vector control (pBAD-ctr). Bars represent mean of biologically independent replicates ± SEM, n = 3.

b Western analysis of RbmA-3xFLAG levels in *V. cholerae* wild-type and *vadR* mutant strains carrying either an empty control plasmid (pCtr) or a constitutive *vadR* overexpression plasmid. Cells were grown at 30 °C without agitation. Whole cell protein fractions were harvested at OD_{600} = 0.4. Bars indicate mean of biologically independent replicates ± SD, n = 3. Statistical significance was determined using one-way ANOVA and post-hoc Holm-Sidak test. Source data underlying panels **a** and **b** are provided as a Source Data file.

Supplementary Figure 3

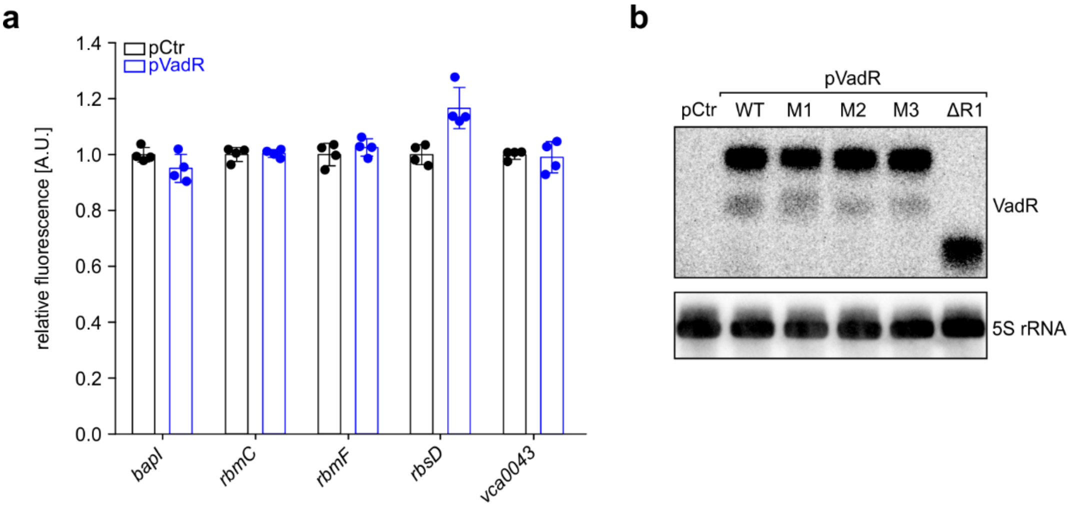

Supplementary Figure 3: Potential VadR target genes and expression of VadR variants

a VadR target genes that do not display post-transcriptional regulation in *E. coli*. Fluorescence intensities of *E. coli* strains carrying the gene-specific reporter and the control plasmid (pCtr) were set to 1. Bars show mean of biologically independent replicates ± SD, *n* = 4.

b Northern analysis confirms similar expression levels of all plasmid-borne VadR variants used in this study. RNA was obtained from *E. coli* cells at OD_{600} = 1.0, which were overexpressing the indicated *vadR* variants. The experiment was done in two biological replicates. Source data underlying panels **a** and **b** are provided as a Source Data file.

Supplementary Figure 4

a

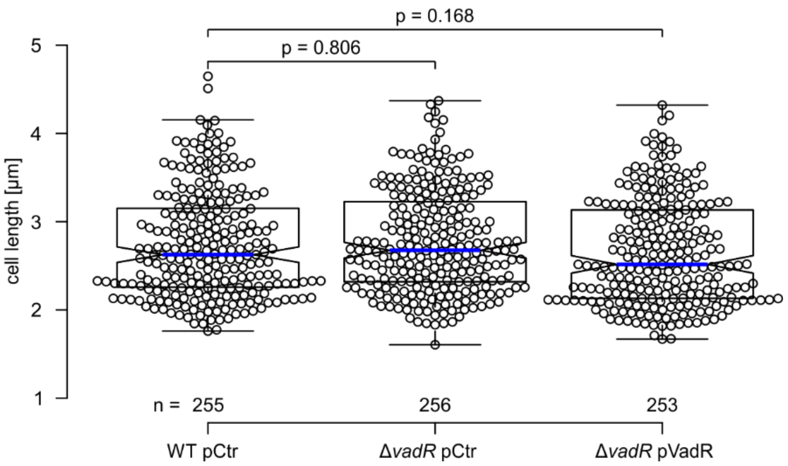

b

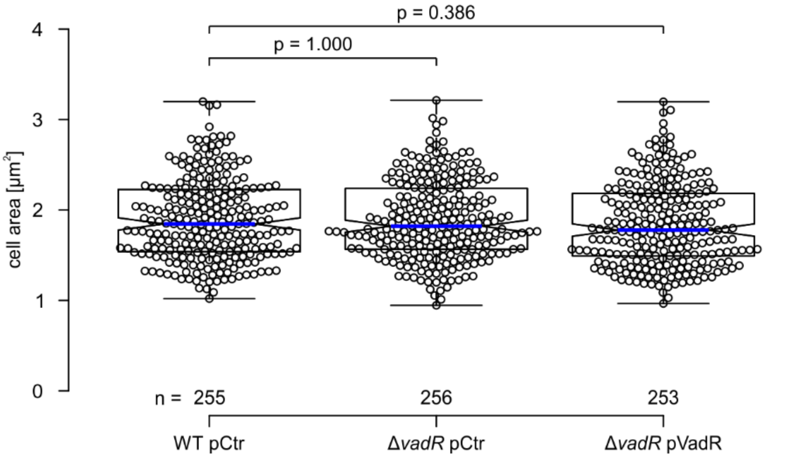

Supplementary Figure 4: VadR does not affect the length and area of *V. cholerae* cells

a-b Analysis of cell length (**a**) and cell area (**b**) in –Cef samples of Fig. 4b. A blue line indicates the median, boxes represent 25th and 75th percentiles, whiskers represent 5th and 95th percentiles and notches indicate 95% confidence intervals for each median. *n* of each set is listed above the x-axis over three independent experiments. Statistical significance was determined using one-sided Kruskal-Wallis test and post-hoc Dunn's test. Source data underlying panels **a** and **b** are provided as a Source Data file.

Supplementary Figure 5

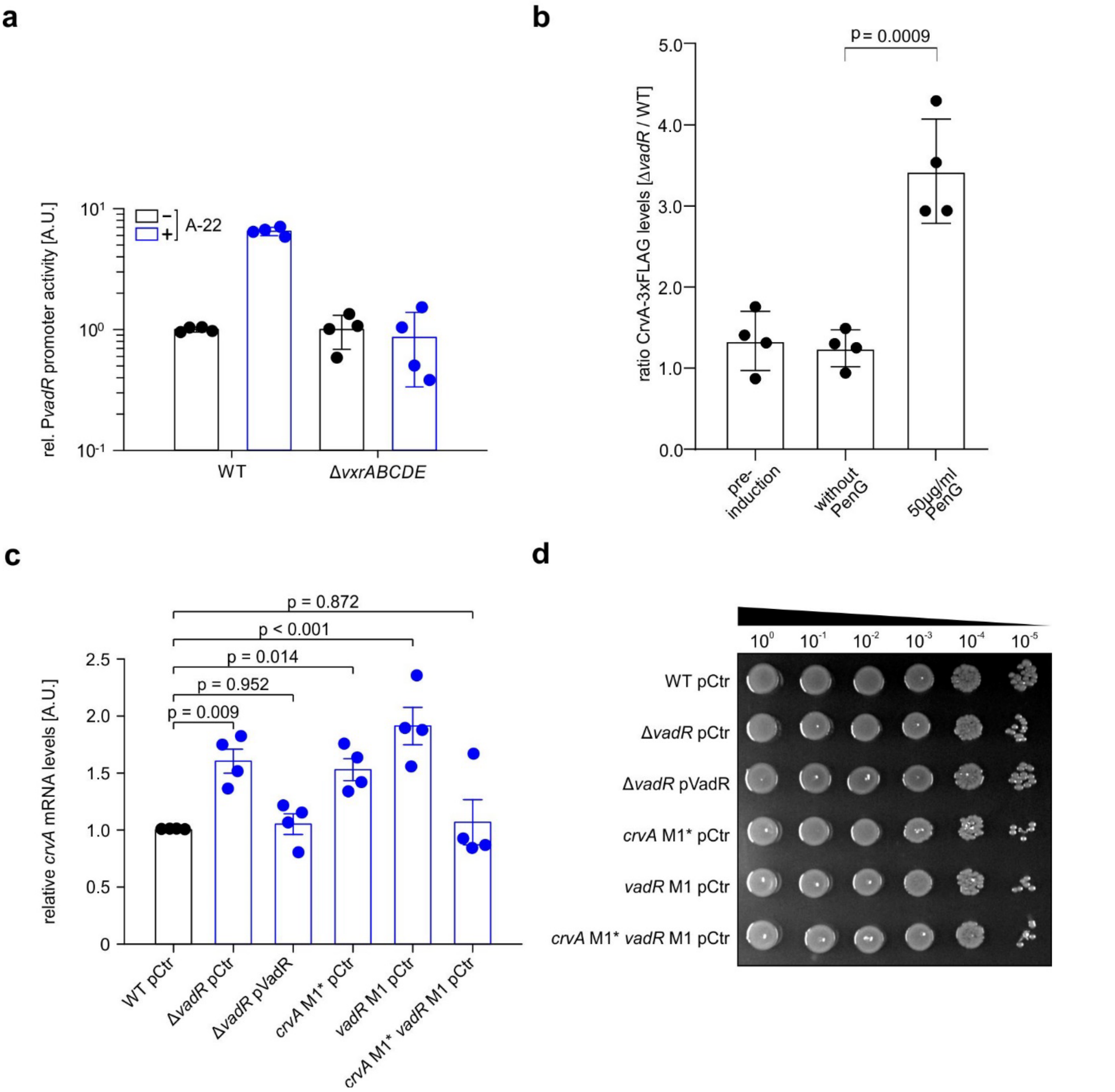

Supplementary Figure 5: The VadR promoter responds to A22 treatment and *V. cholerae* depends on tight *crvA* regulation to overcome Penicillin G stress

a *V. cholerae* wild-type and *vxrABCDE* mutant strains were grown to OD_{600} = 0.2. Cultures were split and one set was treated with A-22 (10 µg/ml final conc.), while the other set received the same volume of water as mock treatment. VadR promoter activities in both sets were measured after 3h using a fluorescent transcriptional reporter. Promoter activities of mock-treated strains were set to 1. Bars represent mean of biologically independent replicates ± SD, n = 4.

b Quantification of CrvA-3xFLAG protein levels in *V. cholerae* wild-type and *vadR*-deficient cells. Strains were grown to an OD_{600} of 0.2 (pre-induction), cultures were split, and one set was treated with penicillin G (50 µg mL^{-1} final conc.) for 3h. Total protein samples of the indicated strains were harvested and tested by Western blot analysis. CrvA-3xFLAG protein

levels in Δ*vadR* were normalized to wild-type levels. Bars show mean of biologically independent replicates ± SD, *n* = 4. Statistical significance was determined using one-way ANOVA and post-hoc Sidak tests.

c The indicated *V. cholerae* strains (x-axis) were grown to an OD_{600} of 0.2 and treated with penicillin G (50 µg mL^{-1} final conc.) for 30 min. Total RNA was isolated and analyzed for *crvA* expression by qRT-PCR. Bars represent mean of biologically independent replicates ± SEM, *n* = 4, relative to *V. cholerae* wild-type. Statistical significance was determined using $\log_{10}$-transformed values for one-way ANOVA and post-hoc Holm-Sidak test.

d The indicated *V. cholerae* strains (y-axis) were grown to OD_{600} = 0.4 + 3 h and assayed for CFUs by spotting serial dilutions on agar plates. Source data underlying panels **a-d** are provided as a Source Data file.

Supplementary Figure 6

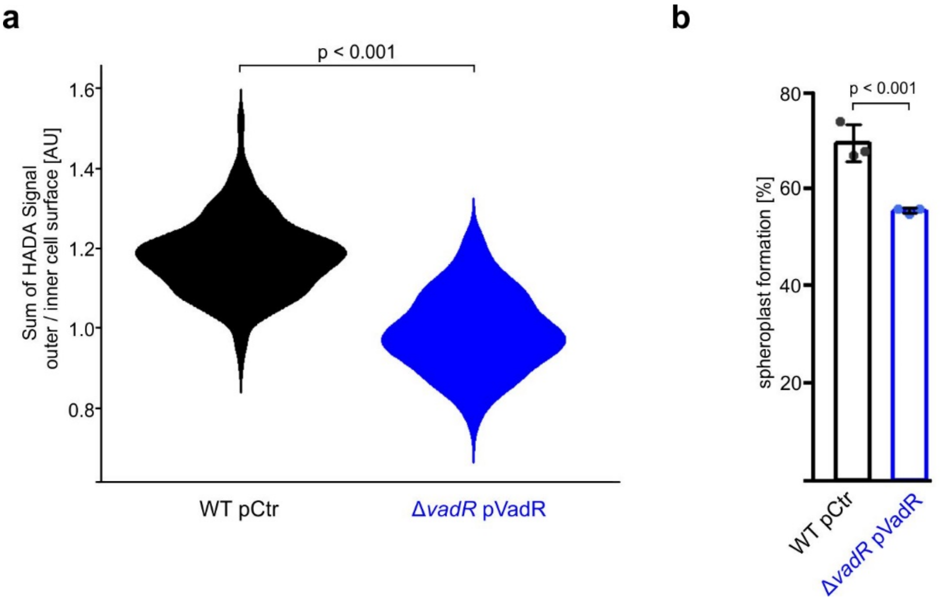

Supplementary Figure 6: Microscopic analysis of *V. cholerae* cells using HADA labelling. a *V. cholerae* wild-type pCtrl and Δ*vadR* pVadR strains were grown to an OD_{600} of 0.3 and peptidoglycan was stained with HADA. The HADA-signal of nascent peptidoglycan serves for an image-based comparison of the outer versus the inner curvature of bent cells by measuring the two cell halves along the centerline separately. *n* = 231 (Δ*vadR* pVadR) and 267 (wild-type pCtrl) over three independent experiment. Statistical significance was determined using one-sided Kruskal-Wallis rank sum test. **b** Spheroplast formation of penicillin G-treated *V. cholerae* cells. *V. cholerae* wild-type pCtrl and *ΔvadR* pVadR strains were grown to an OD_{600} of 0.4 and peptidoglycan was stained with HADA followed by Penicillin G (50 µg mL^{-1} final conc.; 15 min) treatment. Spheroplast formation of individual cells was determined using fluorescent microscopy. The population mean of spheroplast-forming cells is shown. *n* = 3 with 100 analyzed cells per independent experiment. Statistical significance was determined using one-way ANOVA and post-hoc Holm-Sidak test. Source data underlying panels **a** and **b** are provided as a Source Data file.

Supplementary Figure 7

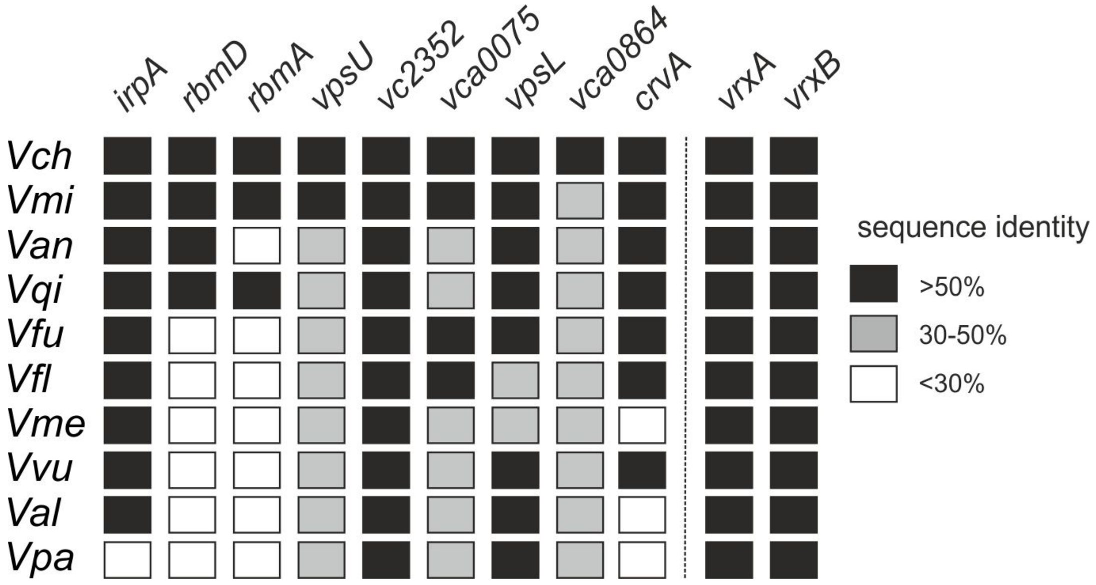

Supplementary Figure 7: Conservation of VadR targets genes among different *Vibrio* species. The presence of *VxrAB* and genes post-transcriptionally regulated by VadR (Fig. 3a) in other *Vibrio* species was analysed by comparing the protein sequences using KEGG[2]. *Vch*: *Vibrio cholerae*, *Vmi*: *Vibrio mimicus*, *Van*: *Vibrio anguillarum*, *Vqi*: *Vibrio qinghaiensis*, *Vfu*: *Vibrio furnissii*, *Vfl*: *Vibrio fluvialis*, *Vme*: *Vibrio mediterranei*, *Vvu*: *Vibrio vulnificus*, *Val*: *Vibrio alginolyticus*, *Vpa*: *Vibrio parahaemolyticus*.

Supplementary Materials and Methods

Plasmid construction

All plasmids and DNA oligonucleotides used in this study are listed in Supplementary Table 3 and Supplementary Table 4, respectively. If not stated otherwise, all insert fragments were amplified from genomic DNA of *V. cholerae* C6706. The backbone for the overexpression plasmids pNP-001/003-006/008-010/013 was linearized with KPO-0092/1023 using pEVS143 as a PCR template. For the amplifications of the inserted sRNAs, the following combinations of oligonucleotides were used: KPO-1003/1004 (pNP-001), KPO-1024/1025 (pNP-003), KPO-1005/1006 (pNP-004), KPO-1015/1016 (pNP-005), KPO-1021/1022 (pNP-006), KPO-1009/1010 (pNP-008), KPO-1219/1220 (pNP-009), KPO-1001/1002 (pNP-010), and KPO-1017/1018 (pNP-013). Subsequently, linearized vector and sRNA inserts were treated with XbaI restriction enzyme and fused by ligation. The construction of overexpression plasmids pLS-014-020, pRH-005, and pSG-001/002 was achieved by Gibson assembly. pEVS143 backbone was linearized using KPO-0092/1397 (pLS-014-020) or KPO-0092/1023 (pRH-005, pSG-001/002). sRNA insert sequences were amplified using KPO-5835/5836 (pLS-014), KPO-5837/5838 (pLS-015), KPO-5841/5842 (pLS-016), KPO-5843/5844 (pLS-017), KPO-5845/5846 (pLS-018), KPO-5847/5848 (pLS-019), KPO-5849/5850 (pLS-020), KPO-1226/1227 (pRH-005), KPO-1858/1859 (pSG-001), and KPO-1860/1861 (pSG-002). Further, Gibson assembly was used to generate the inducible overexpression plasmids pMD-097, pNP-019, and pNP-123-127. For these plasmids, pMD-004 served as backbone and was linearized using KPO-0196/1397 (pMD-097 and pNP-019) or KPO-0196/1488 (pNP-123-127). Amplification of insert genes were achieved with oligonucleotide combinations KPO-2554/2555 (pMD-097), KPO-1400/1401 (pNP-019), KPO-4852/4918 (pNP-123), KPO-4852/4919 (pNP-125), KPO-4852/4920 (pNP-126), and KPO-4852/4921 (pNP-127). Plasmid pNP-124 was assembled from two insert fragments, which were amplified with oligonucleotides KPO-4852/4853 and KPO-4854/4855, respectively. Plasmids pEE-007, pLS-026-028, and pMS-001-002 were generated by oligonucleotide-directed mutagenesis of pNP-005 (pEE-007) and pAE-002 (pLS-026-028, pMS-001-002), using KPO-4098/4099 (pEE-007), KPO-5981/5982 (pLS-026), KPO-5983/5984 (pLS-027), KPO-5985/5986 (pLS-028), KPO-6472/6473 (pMS-001), and KPO-6474/6475 (pMS-002). The promoter region of *vadR* was amplified using KPO-1906/1907 and KPO-4410/4411 for plasmids pAE-002 and pNP-122, respectively. To generate pAE-002, the obtained fragment and the pCMW-1C vector were digested with SphI and SalI enzymes and fused by ligation. Likewise, pNP-122 was obtained by ligation after treating insert and pBBR1-MCS5-lacZ equally with restriction enzymes SpeI and SalI. 5' UTRs and initial coding sequences for the construction of the translational reporter plasmids pNP-064/070-073, pRG-011-013 and pRH-090/092 were amplified using KPO-1720/1721 (pNP-064), KPO-

2067/2068 (pNP-070), KPO-2069/2070 (pNP-071), KPO-2071/2072 (pNP-072), KPO-2065/2066 (pNP-073), KPO-3735/3736 (pNP-113), KPO-3739/3740 (pNP-114), KPO-3737/3738 (pNP-115), KPO-2383/2384 (pRG-011), KPO-2385/2386 (pRG-012), KPO-2389/2390 (pRG-013), KPO-5534/5535 (pRH-090), and KPO-5538/5539 (pRH-092). Restriction digests of the amplified fragments and the pXG10-SF vector were conducted using NsiI and NheI enzymes. Inserts and vectors were combined by ligation. Plasmid pMH-039 was generated by Gibson assembly, using KPO-1702/1703 to linearize the pXG10-SF vector and KPO-1801/2803 to amplify the insert fragment, respectively. To build the suicide plasmid pNP-133, flanking regions of the *vadR* locus were amplified using KPO-1294/1295 and KPO-1296/1297. The two fragments were combined by overlap PCR with KPO-1298/1299. Restriction digest of the obtained insert fragment and of the pKAS32 vector using KpnI and ArvII enzymes and subsequent ligation, yielded the functional plasmid. To build plasmid pRH-093, pNP-133 was linearized with KPO-5550/5551. The required insert was amplified from pNP-117 using KPO-5548/5549. Gibson assembly of both parts resulted in pRH-093. Plasmids pNP-128/132/134/135 and pRH-099 were obtained by Gibson assembly, using a pKAS32 vector, which was linearized with KPO-0267/0268. The single insert fragment of pNP-128 was amplified with KPO-5456/5457. The flanking regions of the *crvA* gene and the *vxrABCDE* operon were amplified using the two oligonucleotide combinations KPO-5450/5451, KPO-5452/5453 (pNP-134) and KPO-4621/4622, KPO-4625/4626 (pNP-135), respectively. To introduce a *crvA-3xFLAG* construct onto the chromosome of *V. cholerae*, plasmid pNP-132 was designed. The corresponding flanking regions were amplified using KPO-5442/5443 and KPO-5446/5447. Oligonucleotides KPO-5444/5445 were used to amplify the 3xFLAG epitope from template plasmid pRH-030. The *araC*-P_{BAD} insert of pRH-099 was amplified from pMD-004 using KPO-4529/0196. Flanking regions of the *crvAB* promoter were amplified using oligos KPO-6013/6014 and KPO-6015/6016, respectively. Plasmid pNUT1403 was generated by ligating the *vadR* promoter fused to *mruby2* gene, amplified with oligo pair KDO-0626 and KDO1721, at XbaI-SphI restriction site of pNUT542 plasmid. *mRuby2* gene was used from pNUT883 and *vadR* promoter region was amplified from plasmid pAE-002. All mutations for compensatory base pair exchanges were introduced by oligonucleotide-directed mutagenesis using the oligonucleotides listed in Supplementary Table 4, and the respective parental plasmids as a template.

Construction of *V. cholerae* mutant strains

All strains used in this study is are listed in Supplementary Table 2. *V. cholerae* C6706 was used as the wild-type strain in this study. *V. cholerae* mutant strains were generated as described previously[3]. Conjugal transfer was used to introduce plasmids into *V. cholerae* from

E. coli S17λpir donor strains. Transconjugants were selected using appropriate antibiotics, and 50 U mL^{-1} polymyxin B was used to select against *E. coli* donor strains.

Transcript stability experiments

Stability of VadR was determined as described previously[4]. Briefly, biological triplicates of *V. cholerae* wild-type (KPS-0014) and Δ*hfq* (KPS-0054) strains were grown to OD_{600} of 0.2and transcription was terminated by addition of 250 µg mL^{-1} rifampicin. Transcript levels were probed and quantified using Northern blot analysis.

Genetic screen for transcriptional regulators of the *vadR* promoter

A plasmid library, expressing *V. cholerae* genomic fragments[5], was screened for activation of *vadR* promoter (P*vadR*) activity. To this end, *lacZ*-deficient *E. coli* BW25113 strains, harboring pNP-122, were transformed with pZach library plasmids. Transformants were selected on LB plates, containing the respective antibiotics and 20 µg mL^{-1} 5-Brom-4-chlor-3-indoxyl-β-D-galactopyranosid (X-gal). 23,000 colonies (representing ~11-fold coverage) were monitored for ß-galactosidase activity.

ß-galactosidase reporter assays

E. coli BW25113 strains harboring the pZ genomic fragment expression plasmids and pNP-122 were grown to OD_{600} of 1.5 in LB. Cells were resuspended in Z-buffer to yield 1.0 OD_{600} mL^{-1}. Cells were lysed by addition of 75 µL chloroform and 50 µL 0.1% SDS and vortexing. Lysates were centrifuged (16.000 x g, 5 min) and the resulting supernatant treated with *O*-Nitrophenyl-β-D-galactopyranoside (ONPG). The reactions were stopped by addition of sodium carbonate (Na_2CO_3). The specific activities were obtained by measuring absorbance at OD_{420}, OD_{550}, and OD_{600} using a Spark 10M plate reader (Tecan). ß-galactosidase activity was deduced from the values by calculating Miller units.

Quantitative real-time PCR

Total RNA was isolated using the SV Total RNA Isolation System (Promega), according to the manufacturer's instructions. qRT-PCR was performed using the Luna Universal One-Step RT-qPCR Kit (New England BioLabs) and the MyiQ™ Single-Color Real-Time PCR Detection System (Bio-Rad). *recA* was used as a reference gene.

Analysis of VxrB-HIS ChIP-seq data

The raw data of the VxrB-HIS ChIP experiment conducted by Shin et al.[1] was obtained from Gene Expression Omnibus (GEO) under the accession number GSE135009. The read files were imported into CLC Genomics Workbench v11 (Qiagen) and trimmed for quality using

default parameters. Reads were mapped to the *V. cholerae* reference genome (NCBI accession numbers: NC_002505.1 and NC_002506.1) using the “RNA-Seq Analysis” tool with default parameters.

RNA *in vitro* analysis

A DNA template carrying the T7 promoter for *in vitro* synthesis of RNA was prepared by PCR using oligonucleotides KPO-5083 and KPO-5084. 200 ng of template DNA were *in vitro* transcribed using the AmpliScribe T7-Flash transcription kit (Epicentre) following the manufacturer’s recommendations. RNA size and integrity were verified on denaturing polyacrylamide gels. For 5’ end labelling, 20 pmol of RNA were dephosphorylated using 10 units of calf alkaline phosphatase (NEB), followed by P:C:I extraction and ethanol precipitation of RNA. Dephosphorylated RNA was incubated with [^{32}P]-γATP (20 µCi) and 1 unit of polynucleotide kinase (NEB) for 1 h at 37 °C. Unincorporated nucleotides were removed using Microspin G-50 Columns (GE Healtcare). Labelled RNA was loaded on a 6%/7 M urea gel, cut from the gel, eluted overnight at 4 °C in RNA elution buffer (0.1 M sodium acetate, 0.1% SDS, 10 mM EDTA), and recovered by P:C:I extraction.

RNA structure probing was carried out as described previously[6] with few modifications. In brief, for 0.4 pmol 5′-end-labelled VadR sRNA was denatured, quickly chilled on ice and supplemented with 1x structure buffer (0.01 M Tris [pH 7], 0.1 M KCl, 0.01 M $MgCl_2$) and 1 µg yeast RNA. Samples were incubated at 37°C, and treated with RNase T1 (0.1 U; Ambion no. AM2283) for 60,120 and 180 sec, or with lead(II) acetate (final concentration, 5 mM; Sigma no. 316512) for 15, 30 and 90 sec.

Reactions were stopped by the addition of 2 vol. stop/precipitation buffer (1 M guanidinium thiocyanate, 0.167% N-lauryl-sarcosine, 10 mM DTT, 83% 2-propanol). RNA was precipitated for 2 h at -20 °C, and collected by centrifugation (30 min, 4 °C, 13.000 rpm). Samples were dissolved in GLII loading buffer and separated on 10% polyacrylamide sequencing gels.

Fluorescence microscopy

V. cholerae strains were cultivated in LB and stained with 50 µM HADA for 5 min at room temperature. Afterwards, the cells were pelleted, washed in 1x PBS, and fixed in 2.5% paraformaldehyde in 1x PBS. To visualize spheroplast formation HADA-stained cells were cultured in LB without any washing steps in the presence of 50 µg/ml penicillin G for 15 min, pelleted, washed in 1xPBS, and fixed in 2.5% paraformaldehyde in 1xPBS. Membrane staining was conducted by addition of 0.5 mg mL^{-1} nile red. Phase contrast imaging was performed on a Zeiss Axio Imager M1 microscope equipped with EC Plan Neofluar 100x/ 1.3 Oil Ph3 objective (Zeiss). For additional magnification and imaging of fluorescent dyes, a 2.5 x optovar

and appropriate filter sets were used. Image acquisition was conducted with the AxioVision software-package (Zeiss).

Supplementary Table 1 Genes differentially regulated by vadR pulse expression

ID	Gene	Description#	Fold change*
vc0932	*rbmE*	uncharacterized protein	-4.70
vc0934	*vpsL*	capsular polysaccharide biosynthesis glycosyltransferase	-4.61
vc0933	*rbmF*	uncharacterized protein	-4.57
vc0928	*rbmA*	rugosity and biofilm structure modulator A	-3.58
vc0935	*vpsM*	polysaccharide biosynthesis protein	-3.48
vc0936	*vpsN*	polysaccharide biosynthesis/export protein	-3.23
vc0917	*vpsA*	UDP-N-acetylglucosamine 2-epimerase	-3.19
vc0919	*vpsC*	serine O-acetyltransferase	-3.03
vc0918	*vpsB*	UDP-N-acetyl-D-mannosaminuronic acid dehydrogenase	-2.92
vc0916	*vpsU*	tyrosine-protein phosphatase	-2.83
vc0931	*rbmD*	hypothetical protein	-2.71
vca0043		hypothetical protein	-2.55
vca0864		methyl-accepting chemotaxis protein	-2.53
vc0920	*vpsD*	polysaccharide biosynthesis protein	-2.47
vc1888	*bap1*	extracellular matrix protein	-2.38
vc0937	*vpsO*	polysaccharide biosynthesis transport protein	-2.38
vc1264	*irpA*	iron-regulated protein A	-2.34
vc0938	*vpsP*	polysaccharide biosynthesis protein	-2.14
vc2352		concentrative nucleoside transporter, CNT familiy	-1.97
vca0075		hypothetical protein	-1.89
vca1075	*crvA*	hypothetical protein	-1.81
vc0930	*rbmC*	rugosity and biofilm structure modulator C	-1.79
vca0044		pseudogene	-1.77
vca0074		diguanylate cyclase	-1.77
vc0018	*ibpA*	molecular chaperone IbpA	-1.76
vca0129	*rbsC*	ribose transport system permease protein	1.81
vca0128	*rbsA*	ribose transport system ATP-binding protein	1.96
vca0127	*rbsD*	D-ribose pyranase	2.22

#Description is based on the annotation at KEGG (https://www.genome.jp/kegg)

*Fold change is based on transcriptomic analysis of pBAD-derived *vadR* expression using RNA-seq. Genes with a fold-change of at least 1.75-fold in either condition and a FDR adjusted p-value ≤ 0.001 were considered to be differentially expressed.

Supplementary **Table 2** Bacterial strains used in this study

**Strain**	**Relevant markers/ genotype**	**Reference/ source**
V. cholerae		
KPS-0014	Wild-type C6706	[7]
KPS-0053	Δ*hapR* C6706	[4]
KPS-0054	Δ*hfq* C6706	[8]
KPVC-10126	Δ*vadR* C6706	This study
KPVC-12430	Δ*vxrABCDE* C6706	This study
KPVC-12817	Δ*crvA* C6706	This study
KPVC-12912	*crvA* M1* C6706	This study
KPVC-12913	*crvA::crvA-3xFLAG* C6706	This study
KPVC-12914	Δ*vadR crvA::crvA-3xFLAG* C6706	This study
KPVC-13214	*vadR* M1 C6706	This study
KPVC-13215	*vadR* M1 *crvA* M1* C6706	This study
KPVC-13223	*rbmA::rbmA-3xFLAG, rbmC::rbmC-3xFLAG, bapI::bapI-3xFLAG* C6706	This study
KPVC-13384	P_{crvAB}::*araC*-P_{BAD} C6706	This study
KPVC-13439	Δ*hapR* Δ*rbmA* C6706	This study
E. coli		
BW25113	*lacI*$^{+}$ *rrnB*$_{T14}$ Δ*lacZ*$_{WJ16}$ *hsdR514* Δ*araBAD*$_{AH33}$ Δ*rhaBAD*$_{LD78}$ *rph-1* Δ*(araB–D)567* Δ*(rhaD–B)568* Δ*lacZ4787(::rrnB-3) hsdR514 rph-1*	[9]
TOP10	*mcr*AΔ*(mrr-hsd*RMS-*mcr*BC) Φ80*lac*ZΔM15Δ*lac*X74*deo*R*rec*A1 *ara*D139Δ*(ara-leu)*7697 *gal*U *gal*K *rps*L *end*A1 *nup*G	Invitrogen
S17λpir	Δ*lacU*169 (Φ*lacZ*ΔM15), *recA*1, *endA*1, *hsdR*17, *thi*-1, *gyrA*96, *relA*1, λ*pir*	[10]

Supplementary Table 3 Plasmids used in this study

Plasmid trivial name	Plasmid stock name-	Plasmid backbone	Relevant fragment	Comment	Origin*, marker	Plasmid copy number per cell	Reference
pBBR1MCS 5-5-lacZ		pBBR1MCS	*lacZ*	Promoterless plasmid for transcriptional reporters	pBBR1, $Gent^R$	15-40[11]	[11]
P*vadR-mKate2*	pAE-002	pCMW-1C	P*vadR-mKate2*	*vadR* transcriptional reporter plasmid	p15A, Cm^R	20-30	This study
pCMW-1C	pCMW-1C	pCMW-1C	Cm^R cassette	Promoterless plasmid for transcriptional reporters	p15A, Cm^R	20-30	[12]
pCtr	pCMW-1K	pCMW-1K	Kan^R cassette	Control plasmid	p15A, Kan^R	20-30	[13]
pKAS32-Δ*rbmA*	pCN-007	pKAS32	up-/downstream flanks of *rbmA*	suicide plasmid for *rbmA* knockout	R6K, Amp^R	suicide plasmid	[14]
pKAS32-*rbmA-3xFLAG*	pCN-018	pKAS32	3xFLAG	*rbmA-3xFLAG* allelic replacement	R6K, Amp^R	suicide plasmid	[14]
pKAS32-*rbmC-3xFLAG*	pCN-019	pKAS32	3xFLAG	*rbmC-3xFLAG* allelic replacement	R6K, Amp^R	suicide plasmid	[14]
pKAS32-*bapl-3xFLAG*	pCN-020	pKAS32	3xFLAG	*bap1-3xFLAG* allelic replacement	R6K, Amp^R	suicide plasmid	[15]
pVadRΔR1	pEE-007	pEVS143	*vadR ΔR1*	*vadR ΔR1* expression plasmid	p15A, Kan^R	20-30	This study
pEVS143	pEVS143	pEVS143	Ptac promoter	Constitutive over-expression plasmid	p15A, Kan^R	20-30	[4]
pKAS32	pKAS32	pKAS32		suicide plasmid for allelic exchange	R6K, Amp^R	suicide plasmid	[16]
pVcr025	pLS-014	pEVS143	*vcr025*	*vcr025* expression plasmid	p15A, Kan^R	20-30	This study
pVcr062	pLS-015	pEVS143	*vcr062*	*vcr062* expression plasmid	p15A, Kan^R	20-30[#]	This study
pVcr058	pLS-016	pEVS143	*vcr058*	*vcr058* expression plasmid	p15A, Kan^R	20-30	This study
pVcr071	pLS-017	pEVS143	*vcr071*	*vcr071* expression plasmid	p15A, Kan^R	20-30	This study
pVcr067	pLS-018	pEVS143	*vcr067*	*vcr067* expression plasmid	p15A, Kan^R	20-30	This study
pVcr094	pLS-019	pEVS143	*vcr094*	*vcr094* expression plasmid	p15A, Kan^R	20-30	This study
pVcr099	pLS-020	pEVS143	*vcr099*	*vcr099* expression plasmid	p15A, Kan^R	20-30	This study
pΔ*site1-mKate2*	pLS-026	pCMW-1C	P*vadR*Δ*site1-mKate2*	*vadR* transcriptional reporter plasmid	p15A, Cm^R	20-30	This study

pΔ*site2-mKate2*	pLS-027	pCMW-1C	P*vadRΔsite2-mKate2*	*vadR* transcriptional reporter plasmid	p15A, Cm^R	20-30	This study
pΔ*site3-mKate2*	pLS-028	pCMW-1C	P*vadRΔsite3-mKate2*	*vadR* transcriptional reporter plasmid	p15A, Cm^R	20-30	This study
pΔ*spacer1/2-mKate2*	pMS-001	pCMW-1C	P*vadRΔsite2-mKate2*	*vadR* transcriptional reporter plasmid	p15A, Cm^R	20-30	This study
pΔ*spacer2/3-mKate2*	pMS-002	pCMW-1C	P*vadRΔsite2-mKate2*	*vadR* transcriptional reporter plasmid	p15A, Cm^R	20-30	This study
pBAD	pMD-004	pBAD-1K		Control plasmid	p15A, Kan^R	20-30	[12]
pVcr084	pMD-097	pEVS143	*vcr084*	*vcr084* expression plasmid	p15A, Kan^R	20-30	This study
p*vca0864-gfp*	pMH-039	pXG10-1C	*vca0864-gfp*	Translational reporter *vca0864-gfp*	pSC101*, Cm^R	3-4	This study
pVcr002	pNP-001	pEVS143	*vcr002*	*vcr002* expression plasmid	p15A, Kan^R	20-30	This study
pVcr036	pNP-003	pEVS143	*vcr036*	*vcr036* expression plasmid	p15A, Kan^R	20-30	This study
pVcr043	pNP-004	pEVS143	*vcr043*	*vcr043* expression plasmid	p15A, Kan^R	20-30	This study
pVadR	pNP-005	pEVS143	*vadR*	*vadR* expression plasmid	p15A, Kan^R	20-30	This study
pVcr079	pNP-006	pEVS143	*vcr079*	*vcr079* expression plasmid	p15A, Kan^R	20-30	This study
pVcr034	pNP-008	pEVS143	*vcr034*	*vcr034* expression plasmid	p15A, Kan^R	20-30	This study
pVcr082	pNP-009	pEVS143	*vcr082*	*vcr082* expression plasmid	p15A, Kan^R	20-30	This study
pVcr092	pNP-010	pEVS143	*vcr092*	*vcr092* expression plasmid	p15A, Kan^R	20-30	This study
pVcr045	pNP-013	pEVS143	*vcr045*	*vcr045* expression plasmid	p15A, Kan^R	20-30	This study
pBAD-*vadR*	pNP-019	pBAD-1K	P_{BAD}-*vadR*	Inducible *vadR* expression plasmid	p15A, Kan^R	20-30	This study
p*rbmC-gfp*	pNP-064	pXG10-1C	*rbmC-gfp*	Translational reporter *rbmC-gfp*	pSC101*, Cm^R	3-4	This study
p*vpsU-gfp*	pNP-070	pXG10-1C	*vpsU-gfp*	Translational reporter *vpsQ-gfp*	pSC101*, Cm^R	3-4	This study
p*rbmA-gfp*	pNP-071	pXG10-1C	*rbmA-gfp*	Translational reporter *rbmA-gfp*	pSC101*, Cm^R	3-4	This study
p*rbmD-gfp*	pNP-072	pXG10-1C	*rbmD-gfp*	Translational reporter *rbmD-gfp*	pSC101*, Cm^R	3-4	This study
p*vpsL-gfp*	pNP-073	pXG10-1C	*vpsL-gfp*	Translational reporter *vpsL-gfp*	pSC101*, Cm^R	3-4	This study
p*irpA-gfp*	pNP-113	pXG10-1C	*irpA-gfp*	Translational reporter *irpA-gfp*	pSC101*, Cm^R	3-4	This study

p*vc2352-gfp*	pNP-114	pXG10-1C	*vc2352-gfp*	Translational reporter *vc2352-gfp*	pSC101*, Cm^R	3-4	This study
p*vca0043-gfp*	pNP-115	pXG10-1C	*vca0043-gfp*	Translational reporter *vca0043-gfp*	pSC101*, Cm^R	3-4	This study
p*crvA M1*-gfp*	pNP-116	pXG10-1C	*crvA M1*-gfp*	Translational reporter *crvA* M1-*gfp*	pSC101*, Cm^R	3-4	This study
pVadR M1	pNP-117	pEVS143	*vadR* M1	*vadR* M1 expression plasmid	p15A, Kan^R	20-30	This study
pVadR M3	pNP-118	pEVS143	*vadR* M3	*vadR* M3 expression plasmid	p15A, Kan^R	20-30	This study
p*vpsU M2*-gfp*	pNP-119	pXG10-1C	*vpsU M2*-gfp*	Translational reporter *vpsU* M2-*gfp*	pSC101*, Cm^R	3-4	This study
p*rbmA M1*-gfp*	pNP-120	pXG10-1C	*rbmA M1*-gfp*	Translational reporter *rbmA* M1-*gfp*	pSC101*, Cm^R	3-4	This study
p*vpsL M3*-gfp*	pNP-121	pXG10-1C	*vpsL M3*-gfp*	Translational reporter *vpsL* M3-*gfp*	pSC101*, Cm^R	3-4	This study
P*vadR-lacZ*	pNP-122	pBBR1MCS	P*vadR-lacZ*	*vadR* transcriptional reporter plasmid	pBBR1, Gent^R	15-40[11]	This study
pBAD-*vxrA*	pNP-123	pBAD-1K	*vxrA*	Inducible *vxrA* expression plasmid	p15A, Kan^R	20-30	This study
pBAD-*vxrAB*	pNP-124	pBAD-1K	*vxrAB*	Inducible *vxrAB* expression plasmid	p15A, Kan^R	20-30	This study
pBAD-*vxrABC*	pNP-125	pBAD-1K	*vxrABC*	Inducible *vxrABC* expression plasmid	p15A, Kan^R	20-30	This study
pBAD-*vxrABCD*	pNP-126	pBAD-1K	*vxrABCD*	Inducible *vxrABCD* expression plasmid	p15A, Kan^R	20-30	This study
pBAD-*vxrABCDE*	pNP-127	pBAD-1K	*vxrABCDE*	Inducible *vxrABCDE* expression plasmid	p15A, Kan^R	20-30	This study
pKAS32-Δ*crvA*	pNP-128	pKAS32	*crvA* region	*crvA* region	R6K, Amp^R	suicide plasmid	This study
pKAS32-*crvA* M1*	pNP-129	pKAS32	*crvA* M1*	*crvA* M1* allelic replacement	R6K, Amp^R	suicide plasmid	This study
pKAS32-*crvA-3xFLAG*	pNP-132	pKAS32	*crvA-3xFLAG*	*crvA-3xFLAG* allelic replacement	R6K, Amp^R	suicide plasmid	This study
pKAS32-Δ*vadR*	pNP-133	pKAS32	up-/downstream flanks *vadR*	suicide plasmid for *vadR* knockout	R6K, Amp^R	suicide plasmid	This study
pKAS32-Δ*crvA*	pNP-134	pKAS32	up-/downstream flanks *crvA*	suicide plasmid for *crvA* knockout	R6K, Amp^R	suicide plasmid	This study
pKAS32-Δ*vxrABCDE*	pNP-135	pKAS32	up-/downstream flanks *vxrABCDE*	suicide plasmid for *vxrABCDE* knockout	R6K, Amp^R	suicide plasmid	This study
pVadR M2	pNP-168	pEVS143	*vadR* M2	*vadR* M2 expression plasmid	p15A, Kan^R	20-30	This study
pNUT542	pNUT542	pEVS143	Ptac-*sfGFP*	sfGFP expression plasmid	pSC101, Gent^R	3-4	[17]

pNUT883	pNUT883	pEVS143	*mRuby2*	Promoterless plasmid for transcriptional reporters	p15A, $Gent^R$	20-30	[17]
pNUT1403	pNUT1403	pEVS143	P*vadR*-*mRuby2*	*vadR* transcriptional reporter plasmid	pSC101, $Gent^R$	3-4	This study
p*bap1-gfp*	pRG-011	pXG10-1C	*bap1-gfp*	Translational reporter *bap1-gfp*	pSC101*, Cm^R	3-4	This study
p*crvA-gfp*	pRG-012	pXG10-1C	*crvA-gfp*	Translational reporter *crvA-gfp*	pSC101*, Cm^R	3-4	This study
p*rbmF-gfp*	pRG-013	pXG10-1C	*rbmF-gfp*	Translational reporter *rbmEF-gfp*	pSC101*, Cm^R	3-4	This study
pVcr098	pRH-005	pEVS143	*vcr098*	*vcr098* expression plasmid	p15A, Kan^R	20-30	This study
pKAS32-*aphA-3xFLAG*	pRH-030	pKAS32	3xFLAG	*aphA-3xFLAG* allelic replacement	R6K, Amp^R	suicide plasmid	[16]
p*vca0075-gfp*	pRH-090	pXG10-1C	*vca0075-gfp*	Translational reporter *vca0075-gfp*	pSC101*, Cm^R	3-4	This study
p*rbsD-gfp*	pRH-092	pXG10-1C	*rbsD-gfp*	Translational reporter *rbsD-gfp*	pSC101*, Cm^R	3-4	This study
pKAS32-*vadR* M1	pRH-093	pKAS32	*vadR* M1	*vadR* M1 allelic replacement	R6K, Amp^R	suicide plasmid	This study
pKAS32-*araC*-P_{BAD}	pRH-099	pKAS32	*araC*-P_{BAD}, flanking regions of P_{crvAB}	*araC*-P_{BAD} allelic replacement	R6K, Amp^R	suicide plasmid	This study
pVcr017	pSG-001	pEVS143	*vcr017*	*vcr017* expression plasmid	p15A, Kan^R	20-30	This study
pVcr080	pSG-002	pEVS143	*vcr080*	*vcr080* expression plasmid	p15A, Kan^R	20-30	This study
pXG10-SF	pXG10SF	pXG10-1C	'*lacZ::gfp*	template plasmid for translational reporters	pSC101*, Cm^R	3-4	[18]
pCMW-1C-mKate2	pYH-010	pCMW-1C	mKate2	Promoterless plasmid for transcriptional reporters	P15A, Cm^R	20-30	[9]
pZ1	pZ1		*vxrAB* fragment 1	*vxrAB* fragment 1 expression plasmid	p15A, Cm^R	20-30	This study
pZ2	pZ2		*vxrAB* fragment 2	*vxrAB* fragment 2 expression plasmid	p15A, Cm^R	20-30	This study
pZach	pZND132		*V.ch.* genomic fragments	Genomic fragment expression plasmid	p15A, Cm^R	20-30	[5]

* Plasmids with the origins of replication p15a and pBBR1 were combined in Supplementary Figure 1e. For Figs. 3a, f-i and Supplementary Figure 3a plasmids with the origins of replication p15A / pSC101* were combined. These combinations of origins of replication are compatible (https://blog.addgene.org/plasmid-101-origin-of-replication). Plasmid copy numbers were obtained form [19].

Supplementary Table 4 DNA oligonucleotides used in this study

Sequences are given in 5' → 3' direction; 5' P denotes a 5' monophosphate

ID	Sequence	Description
KDO-0626	TAGCTCCTGAATTCCTAGGCCTG	pNUT1403
KDO-1721	GGGTCTAGAGCGGAGTGACTATAAAAAGGCGC	pNUT1403
KPO-0092	CCACACATTATACGAGCCGA	pNP-001/003-006/008-010/013, pSG001/002, pLS014-020, pRH-005
KPO-0196	GGAGAAACAGTAGAGAGTTGCG	pNP-019/123-127, pMD-097, pRH-099
KPO-0243	TTCGTTTCACTTCTGAGTTCGG	5S-rRNA probe
KPO-0267	TAATAGGCCTAGGATGCATATG	pNP-128/132/133/134/135, pRH-099
KPO-0268	CGTTAACAACCGGTACCTCTA	pNP-128/132/133/134/135, pRH-099
KPO-0331	GAGCCAATCTACAATTCATCAGA	VadR probe
KPO-1001	P-TCACAGAACCGCTGTGACCA	pNP-010
KPO-1002	GTTTTTTCTAGATTGACTACTTCATTCGCCAC	pNP-010
KPO-1003	P-GCAAACACATTGGTAAGATATTAG	pNP-001
KPO-1004	GTTTTTTCTAGATATAACCTGTTCAGAATGTGCT	pNP-001
KPO-1005	P-GTCATCTCGTTAGTCATTACGA	pNP-004
KPO-1006	GTTTTTTCTAGACACTGACAAACCGGTGTTGG	pNP-004
KPO-1009	P-ACTTACTTGGATAAATATGCATTG	pNP-008
KPO-1010	GTTTTTTCTAGAGTATTGTTTGTCTGTCATAAAGTT	pNP-008
KPO-1015	P-AATAGACAACCTTTTGTCCTATC	pNP-005
KPO-1016	GTTTTTTCTAGAATAGAAAGCACTGAGTCAGGA	pNP-005
KPO-1017	P-TTGCCCGCAAGCCACGGC	pNP-013
KPO-1018	GTTTTTTCTAGAAGGCGATTGGTCGTGTTGTT	pNP-013
KPO-1021	P-GTTTGAACCCCGGCGGCT	pNP-006
KPO-1022	GTTTTTTCTAGAAAACCGACTCCTTGCAAGAA	pNP-006
KPO-1023	GTTTTTTCTAGAGGATCCGGTGATTGATTGAG	pNP-001/003-006/008-010/013, pSG001/002, pRH-005
KPO-1024	P-ACCCAAAGGGTAGAGCAAAC	pNP-003
KPO-1025	GTTTTTTCTAGAGAAAACGAAGTAATCTTCACCTT	pNP-003
KPO-1219	P-AGCTTCGCTAGCGAAGAG	pNP-009
KPO-1220	GTTTTTTCTAGAGAATGTTGCGATCAAGTTCG	pNP-009
KPO-1226	TCGTATAATGTGTGGGTAAGGTTAGTGAGAACATTTCT	pRH-005
KPO-1227	ACCGGATCCTCTAGAAGTTTCAAATTTCGTGGACAGC	pRH-005
KPO-1294	GTACATTTTGGTGTGGGAGC	pNP-133
KPO-1295	GCACTGAGTCAGGATTTTGCGTATCGGCGGTTATTCGGTTC	pNP-133
KPO-1296	GCAAAATCCTGACTCAGTGC	pNP-133
KPO-1297	CAAACCCAGCTCTTTAGCTTC	pNP-133
KPO-1298	GTTTTTGGTACCGACGCGAGATTATTTCTTCC	pNP-133
KPO-1299	GTTTTTCCTAGGGATAGTCAGGCCGCTTTCG	pNP-133
KPO-1397	GATCCGGTGATTGATTGAGC	pNP-019, pMD-097, pLS014-020
KPO-1400	CGCAACTCTCTACTGTTTCTCCGAATAGACAACCTTTTGTCCTATC	pNP-019
KPO-1401	GCTCAATCAATCACCGGATCATAGAAAGCACTGAGTCAGGA	pNP-019
KPO-1488	TTTTTTCTAGATTAAATCAGAACGCAG	pNP-123-127

KPO-1702	ATGCATGTGCTCAGTATCTCTATC	pMH-039
KPO-1703	GCTAGCGGATCCGCTGG	pMH-039
KPO-1720	GAGATACTGAGCACATGCATAGGTTGTTATTAGCAATCCGCGATAC	pNP-064
KPO-1721	GAGCCAGCGGATCCGCTAGCCAACGACAAAAGACCGACAGCAAG	pNP-064
KPO-1801	CTGTCACCAATTACGCTGGTTTTTCCTTTTTATTAAC	pMH-039
KPO-1858	TCGGCTCGTATAATGTGTGGGCTAGCGAAAACTATAATCATAAAC	pSG-001
KPO-1859	CTCAATCAATCACCGGATCCGCTTTGATTGAGCAGACGTTG	pSG-001
KPO-1860	TCGGCTCGTATAATGTGTGGGCAAGTCAGTGGTGTTGG	pSG-002
KPO-1861	CTCAATCAATCACCGGATCCGTACTGTCAATATCGACCAC	pSG-002
KPO-1906	GTTTTTGCATGCGCTGCGTGTTGAAAACGATG	pAE-002
KPO-1907	GTTTTTGTCGACCTATTCGTGAAGCAGTGTATC	pAE-002
KPO-2065	GTTTTTATGCATAGATATTTCTATTGATAAAGATGTAGTCTT	pNP-073
KPO-2066	GTTTTTGCTAGCGCTATCAATTAATCGGTAGAAAAATTTAC	pNP-073
KPO-2067	GTTTTTATGCATACTCTGATAATGAGTAGATTGCG	pNP-070
KPO-2068	GTTTTTGCTAGCCTCTGCCATTGGCGAACGA	pNP-070
KPO-2069	GTTTTTATGCATTTAGCCAATGCAATTGTCTTAGATTTG	pNP-071
KPO-2070	GTTTTTGCTAGCATAAGAAGCCGTTGAAAATAACAATGC	pNP-071
KPO-2071	GTTTTTATGCATATGGCATGGCGGAGCAAGTTG	pNP-072
KPO-2072	GTTTTTGCTAGCACTGCCAAGAGGGATTGGTAAC	pNP-072
KPO-2378	GGTAACCCAGAAACTACCACTG	*recA* qRT-PCR
KPO-2379	CACCACTTCTTCGCCTTCTT	*recA* qRT-PCR
KPO-2383	GTTTTTATGCATGCTCTCAGCATATCGTTATTG	pRG-011
KPO-2384	GTTTTTGCTAGCGAATGCGGTGCTTTGAGTC	pRG-011
KPO-2385	GTTTTTATGCATGCTTAGATCTAAAGTTCAAAAAATCAG	pRG-012
KPO-2386	GTTTTTGCTAGCCGATGCAGATACCCATAAAGG	pRG-012
KPO-2389	GTTTTTATGCATAAAGAAATAATATGTATCGTTTATCG	pRG-013
KPO-2390	GTTTTTGCTAGCATTCATGCTAGGAAAAAATGCAATC	pRG-013
KPO-2554	CGCAACTCTCTACTGTTTCTCCTATTACAACAAGAGAGGCTC	pMD-097
KPO-2555	GCTCAATCAATCACCGGATCCAGACGCTACATCAAACTG	pMD-097
KPO-2803	GAGCCAGCGGATCCGCTAGCGACCACCCAACGCAGCAATC	pMH-039
KPO-3613	CTTGATTGGTTGGCGTGTATTG	*vpsL-O* qRT-PCR
KPO-3614	CTTGCCCTTGAGTAGTCATACC	*vpsL-O* qRT-PCR
KPO3615	CTTGTGGCGCACTTTCAATC	*rbmEF* qRT-PCR
KPO3616	GTGGATGACCAACGAGTACAA	*rbmEF* qRT-PCR
KPO-3617	GCTCTTACTGATGGTCGTATGT	*rbmA* qRT-PCR
KPO-3618	CTGCAACGACTTGAAGAGAAAC	*rbmA* qRT-PCR
KPO-3621	TAGTGCTGGCACGCTAAAG	*vpsQA-K* qRT-PCR
KPO-3622	TTGAGTCACTTGCTGGACTG	*vpsQA-K* qRT-PCR
KPO-3623	CTTGGTTGCCGCGTTATTG	*rbmD* qRT-PCR
KPO-3624	GCATAGAAGGCCTGACAGATAC	*rbmD* qRT-PCR
KPO-3625	GAGCTGCAAGGTAAGGGATAC	*vca0043-44* qRT-PCR
KPO-3626	AACTACAGACGGGCACAATC	*vca0043-44* qRT-PCR
KPO-3627	CAGTCCCTATCCGAGCATATTG	*vca0864* qRT-PCR
KPO-3628	GGTAAGCTCCTCTAACCGATAAC	*vca0864* qRT-PCR
KPO-3629	CCGTCTCTTACTGGTTCTTTGG	*bapI* qRT-PCR
KPO-3630	GTGTCACAGGAACGGCATAA	*bapI* qRT-PCR
KPO-3631	CGATCTTGAGTGGATGGAGAAG	*irpA* qRT-PCR
KPO-3632	ATAGCGAGCCCATACCAAAC	*irpA* qRT-PCR
KPO-3633	GCGTGAAAGTAGCGTGTTAGA	*crvA* qRT-PCR

KPO-3634	TTCTGCTTCGTCAGGTATTGG	*crvA* qRT-PCR
KPO-3635	CTGAGCTGTTTGCGGTAATG	*vc2352* qRT-PCR
KPO-3636	CCGCTACCAAGTATTCGATCT	*vc2352* qRT-PCR
KPO-3637	GGCATCGAACATCACGATACA	*vca0074-75* qRT-PCR
KPO-3638	CCATGGCAGTTCAGTGGTAAA	*vca0074-75* qRT-PCR
KPO-3641	TCGGCCATACCGATGAAATC	*rbsDACB* qRT-PCR
KPO-3642	AGTCAGCGCGAGATCAATAC	*rbsDACB* qRT-PCR
KPO-3643	GGTTCTGAGCTATGGAGCTATG	*rbmC* qRT-PCR
KPO-3644	ATCTCAACGATTCCGTCACC	*rbmC* qRT-PCR
KPO-3735	GTTTTTATGCATGAAATAACAAATGATAATAATTTGCAATTC	pNP-113
KPO-3736	GTTTTTGCTAGCCGCTGATGTAGTGAGCGTC	pNP-113
KPO-3737	GTTTTTATGCATAGCGAGTCACCAACTAATTTG	pNP-115
KPO-3738	GTTTTTGCTAGCTTCCAAAGCCACGCGATAAC	pNP-115
KPO-3739	GTTTTTATGCATGCTTAATCGCTCCATTTTGTAAC	pNP-114
KPO-3740	GTTTTTGCTAGCCAGTAGAACTGCGATTCCTAG	pNP-114
KPO-4098	TCGGCTCGTATAATGTGTGGATCTGATGAATTGTAGATTGGCT	pEE-007
KPO-4099	AATAGACAACCTTTTGTCCTATCTGATGAATTGATATGTTTTAAGC	pEE-007
KPO-4250	GAATACTGAACCTTTTGTCCTATCTG	pNP-117
KPO-4251	GTTCAGTATTCCCACACATTATACG	pNP-117
KPO-4252	GTTTCAGTTTCCCACTTTATGTGG	pNP-116
KPO-4253	GGAAACTGAAACTTTTGACAGCTTTG	pNP-116
KPO-4410	GTTTTTTACTAGTGCTGCGTGTTGAAAACGATG	pNP-122
KPO-4411	GTTTTTTGTCGACCTATTCGTGAAGCAGTGTATC	pNP-122
KPO-4529	TATAAGATCATAAAAGACCCTTCATTTATG	pRH-099
KPO-4621	AGAGGTACCGGTTGTTAACGCATCATCAAGTCCACACCACT	pNP-135
KPO-4622	TATCCGGTAAAGAGATATTCGAG	pNP-135
KPO-4625	GAATATCTCTTTACCGGATACACCAAACCTGCTAAAAACACG	pNP-135
KPO-4626	TATGCATCCTAGGCCTATTACGATACCGGTGAAGCTAATGA	pNP-135
KPO-4846	GATTGGCTTTGACCGTCTACT	*ibpA* qRT-PCR
KPO-4847	GCTCGATATTGTATGGAGGGTATC	*iboA* qRT-PCR
KPO-4852	CAACTCTCTACTGTTTCTCCGGATAATGCGTTATAGTTTTTGC	pNP-123-127
KPO-4853	TCAACGAGAAGCAGTGTCTG	pNP-124
KPO-4854	CAGACACTGCTTCTCGTTGAAGATGATAAAAACCTCGCTGAC	pNP-124
KPO-4855	CTGATTTAATCTAGAAAAAAATGATCACGCTTTCATTTTGTAAC	pNP-124
KPO-4918	CTGATTTAATCTAGAAAAAAATCAACGAGAAGCAGTGTCTG	pNP-123
KPO-4919	CTGATTTAATCTAGAAAAAAACTATAGCGGCATATTGTCCAA	pNP-125
KPO-4920	CTGATTTAATCTAGAAAAAAAGAGCCACACTATAAAGAGATG	pNP-126
KPO-4921	CTGATTTAATCTAGAAAAAAAGAAAAATTGGCTACGATTATTACC	pNP-127
KPO-5083	GTTTTTTTTAATACGACTCACTATAGGAATAGACAACCTTTTGTCCT	In-vitro VadR
KPO-5084	AAAAAAAGAGCGAGCTATTTAAAC	In-vitro VadR
KPO-5442	AGAGGTACCGGTTGTTAACGGCTTAGATCTAAAGTTCAAAAAATCAG	pNP-132
KPO-5443	GCTGTCTTTGTTTGGTCTGAG	pNP-132
KPO-5444	TCAGACCAAACAAAGACAGCGACTACAAAGACCATGACGGTG	pNP-132
KPO-5445	ATCGTTGGATTTTTGTGCGGTTACTATTTATCGTCATCTTTGTAGTC	pNP-132
KPO-5446	CCGCACAAAAATCCAACGATTTC	pNP-132
KPO-5447	TATGCATCCTAGGCCTATTAGCAGCAATACTTCAACCGGAG	pNP-132
KPO-5450	AGAGGTACCGGTTGTTAACGGAGCTCAATAAGCGAGGAATTC	pNP-134
KPO-5451	GAAATATGCAAGCTGAGTTTTCC	pNP-134
KPO-5452	AAACTCAGCTTGCATATTTCGTCGGAATTCACAAACCTGTC	pNP-134

KPO-5453	TATGCATCCTAGGCCTATTAGAATGGTCTGATCGGAGGTG	pNP-134
KPO-5456	AGAGGTACCGGTTGTTAACGGAACGTACTTTGATTGGAAAAACC	pNP-128
KPO-5457	TATGCATCCTAGGCCTATTACTTCTTTCGATACGGTGACTTG	pNP-128
KPO-5458	GTTTCAGTTTCCCACTTTATGTGGCTAAAC	pNP-129
KPO-5459	GAAACTGAAACTTTTGACAGCTTTGTAGATAG	pNP-129
KPO-5534	GTTTTTATGCATCAAATAATGATGATTAGCCGTCAA G	pRH-090
KPO-5535	GTTTTTGCTAGCGTTCGATGCCAAAGCGAGAG	pRH-090
KPO-5538	GTTTTTATGCATGTAAACTATTATGTCATCGAAACG	pRH-092
KPO-5539	GTTTTTGCTAGCCACCAAGTAAGAGAGTTCAGAG	pRH-092
KPO-5548	CCGCCGATACACTGCTTCACGAATACTGAACCTTTTGTCCTATC	pRH-093
KPO-5549	GATTTTGCCAAATCGTAGGCAAAAAAAGAGCGAGCTATTTAAACTC	pRH-093
KPO-5550	TTCAGTATTCGTGAAGCAGTGTATCGGCGGTTATTCGGTTC	pRH-093
KPO-5551	CTTTTTTTGCCTACGATTTGGCAAAATCCTGACTCAGTGC	pRH-093
KPO-5552	GAGCGAGCTATTTAAACTCGC	VadR 3' probe
KPO-5692	GTTCAGTAACTTTAAAGGATCTATCATG	pNP-120
KPO-5693	GTTACTGAACCATTTGTTTTTACAACTG	pNP-120
KPO-5696	GTTACCGTATGAAGGTTAAAGGTTTATCAG	pNP-119
KPO-5697	CATACGGTAACTACGCACATGATTTAATATTG	pNP-119
KPO-5698	CAAGGTTTTGTCCTATCTGATGAATTG	pNP-168
KPO-5699	CAAAACCTTGTCTATTCCCACACATTA	pNP-168
KPO-5700	GTGGTATCTGATGAATTGTAGATTGG	pNP-118
KPO-5701	GATACCACAAAAGGTTGTCTATTCC	pNP-118
KPO-5743	GAACCAAAAAAGCAGAATACGCATTAC	pNP-121
KPO-5744	CTTTTTTGGTTCATCACTAGACGCTC	pNP-121
KPO-5835	TCGGCTCGTATAATGTGTGGGCGGGTAAAACGCAACTAATC	pLS014
KPO-5836	GCTCAATCAATCACCGGATCCCACCATTTTATGCTCTAGAAATG	pLS014
KPO-5837	TCGGCTCGTATAATGTGTGGGAGAGGTACATAAGAGTTCAAG	pLS-015
KPO-5838	GCTCAATCAATCACCGGATCCGATGTTTTAGGGATATAAAAATAG	pLS-015
KPO-5841	TCGGCTCGTATAATGTGTGGATATATTTCCCAAAGTGGGAAATAG	pLS-016
KPO-5842	GCTCAATCAATCACCGGATCGGAATTGATATGATGAAGACAGAAA	pLS-016
KPO-5843	TCGGCTCGTATAATGTGTGGAGAATCGTTGCTAATCCTGCG	pLS-017
KPO-5844	GCTCAATCAATCACCGGATCCAATGCTCAGTCGTTTGGGTAT	pLS-017
KPO-5845	TCGGCTCGTATAATGTGTGGCCCGAACAGTCTATTTTGCTATTC	pLS-018
KPO-5846	GCTCAATCAATCACCGGATCCCAATCACATAGTCTGCCTATGC	pLS-018
KPO-5847	TCGGCTCGTATAATGTGTGGAATTTGATTATTCTGAATAACCATTAC	pLS-019
KPO-5848	GCTCAATCAATCACCGGATCGTGACTTGCAACTCCGAGT	pLS-019
KPO-5849	TCGGCTCGTATAATGTGTGGGAACTCAGTAGAATCGCTTAGG	pLS-020
KPO-5850	GCTCAATCAATCACCGGATCGCACCATTTTACCGTGGTTTAG	pLS-020
KPO-5981	GTTTTGTTAAACCTGACAACAGTCTGAC	pLS-026
KPO-5982	GTTTAACAAAACCAACGCCCAGCC	pLS-026
KPO-5983	AAACCAACAGTCTGACATTGAACCGAATAAC	pLS-027
KPO-5984	CTGTTGGTTTAAGTCACAAAACCAACGC	pLS-027
KPO-5985	CAGTCATTGAACCGAATAACCGCCG	pLS-028
KPO-5986	TCAATGACTGTTGTCAGGTTTAAGTCAC	pLS-028
KPO-6013	CAACTCTCTACTGTTTCTCC GCTTAGATCTAAAGTTCAAAAAATC	pRH-099
KPO-6014	TATGCATCCTAGGCCTATTA GTTCGCCCACTGTTTATCTTG	pRH-099
KPO-6015	GGGTCTTTTATGATCTTATA CGTTTTGAAGCAATTTGAGATACC	pRH-099
KPO-6016	AGAGGTACCGGTTGTTAACG GTAGTCACTAGGGTTTTGTCATC	pRH-099
KPO-6472	TTGTG ACT GAC AAC AGT CTG AC	pMS-001

KPO-6473	GTTGTCAGTCACAAAACCAACG	pMS-001
KPO-6474	CTG ACT GAC ATT GAA CCG AAT AAC	pMS-002
KPO-6475	GTCAGTCAGGTTTAAGTCACAAAAC	pMS-002

Supplemental References

1. Shin, J.-H. et al. A multifaceted cellular damage repair and prevention pathway promotes high level tolerance to β-lactam antibiotics. 777375 (2019).
2. Kanehisa, M. & Goto, S. KEGG: kyoto encyclopedia of genes and genomes. *Nucleic Acids Res* **28**, 27-30 (2000).
3. Papenfort, K. et al. A Vibrio cholerae autoinducer-receptor pair that controls biofilm formation. *Nat Chem Biol* **13**, 551-557 (2017).
4. Papenfort, K., Forstner, K.U., Cong, J.P., Sharma, C.M. & Bassler, B.L. Differential RNA-seq of Vibrio cholerae identifies the VqmR small RNA as a regulator of biofilm formation. *Proc Natl Acad Sci U S A* **112**, E766-75 (2015).
5. Donnell, Z. Regulation of cqsA and cqsS in Vibrio cholerae. (Princeton, NJ : Princeton University, 2015).
6. Frohlich, K.S., Papenfort, K., Fekete, A. & Vogel, J. A small RNA activates CFA synthase by isoform-specific mRNA stabilization. *EMBO J* **32**, 2963-79 (2013).
7. Thelin, K.H. & Taylor, R.K. Toxin-coregulated pilus, but not mannose-sensitive hemagglutinin, is required for colonization by Vibrio cholerae O1 El Tor biotype and O139 strains. *Infect Immun* **64**, 2853-6 (1996).
8. Svenningsen, S.L., Tu, K.C. & Bassler, B.L. Gene dosage compensation calibrates four regulatory RNAs to control Vibrio cholerae quorum sensing. *EMBO J* **28**, 429-39 (2009).
9. Datsenko, K.A. & Wanner, B.L. One-step inactivation of chromosomal genes in Escherichia coli K-12 using PCR products. *Proc Natl Acad Sci U S A* **97**, 6640-5 (2000).
10. de Lorenzo, V. & Timmis, K.N. Analysis and construction of stable phenotypes in gram-negative bacteria with Tn5- and Tn10-derived minitransposons. *Methods Enzymol* **235**, 386-405 (1994).
11. Fried, L., Lassak, J. & Jung, K. A comprehensive toolbox for the rapid construction of lacZ fusion reporters. *J Microbiol Methods* **91**, 537-43 (2012).
12. Herzog, R., Peschek, N., Frohlich, K.S., Schumacher, K. & Papenfort, K. Three autoinducer molecules act in concert to control virulence gene expression in Vibrio cholerae. *Nucleic Acids Res* **47**, 3171-3183 (2019).
13. Waters, C.M. & Bassler, B.L. The Vibrio harveyi quorum-sensing system uses shared regulatory components to discriminate between multiple autoinducers. *Genes Dev* **20**, 2754-67 (2006).
14. Nadell, C.D., Drescher, K., Wingreen, N.S. & Bassler, B.L. Extracellular matrix structure governs invasion resistance in bacterial biofilms. *ISME J* **9**, 1700-9 (2015).
15. Yan, J., Sharo, A.G., Stone, H.A., Wingreen, N.S. & Bassler, B.L. Vibrio cholerae biofilm growth program and architecture revealed by single-cell live imaging. *Proc Natl Acad Sci U S A* **113**, E5337-43 (2016).
16. Skorupski, K. & Taylor, R.K. Positive selection vectors for allelic exchange. *Gene* **169**, 47-52 (1996).
17. Singh, P.K. et al. Vibrio cholerae Combines Individual and Collective Sensing to Trigger Biofilm Dispersal. *Curr Biol* **27**, 3359-3366 e7 (2017).
18. Corcoran, C.P. et al. Superfolder GFP reporters validate diverse new mRNA targets of the classic porin regulator, MicF RNA. *Mol Microbiol* **84**, 428-45 (2012).
19. Jahn, M., Vorpahl, C., Hubschmann, T., Harms, H. & Muller, S. Copy number variability of expression plasmids determined by cell sorting and Droplet Digital PCR. *Microb Cell Fact* **15**, 211 (2016).
