# Supplementary material for: RNA-mediated control of cell shape modulates antibiotic resistance in Vibrio cholerae
Source: Nat Commun. 2020 Nov 27;11:6067. doi: 10.1038/s41467-020-19890-8 (PMC7695739; doi:10.1038/s41467-020-19890-8)
Supplement: Supplementary file 2 — Reporting Summary [file 41467_2020_19890_MOESM2_ESM.pdf]

nature research

Corresponding author(s): Kai Papenfort

Last updated by author(s): Nov 4, 2020

Reporting Summary

Nature Research wishes to improve the reproducibility of the work that we publish. This form provides structure for consistency and transparency in reporting. For further information on Nature Research policies, see Authors & Referees and the Editorial Policy Checklist.

Statistics

For all statistical analyses, confirm that the following items are present in the figure legend, table legend, main text, or Methods section.

n/a	Confirmed	
☐	☒	The exact sample size (*n*) for each experimental group/condition, given as a discrete number and unit of measurement
☐	☒	A statement on whether measurements were taken from distinct samples or whether the same sample was measured repeatedly
☐	☒	The statistical test(s) used AND whether they are one- or two-sided<br>*Only common tests should be described solely by name; describe more complex techniques in the Methods section.*
☒	☐	A description of all covariates tested
☐	☒	A description of any assumptions or corrections, such as tests of normality and adjustment for multiple comparisons
☐	☒	A full description of the statistical parameters including central tendency (e.g. means) or other basic estimates (e.g. regression coefficient) AND variation (e.g. standard deviation) or associated estimates of uncertainty (e.g. confidence intervals)
☐	☒	For null hypothesis testing, the test statistic (e.g. *F*, *t*, *r*) with confidence intervals, effect sizes, degrees of freedom and *P* value noted<br>*Give P values as exact values whenever suitable.*
☒	☐	For Bayesian analysis, information on the choice of priors and Markov chain Monte Carlo settings
☒	☐	For hierarchical and complex designs, identification of the appropriate level for tests and full reporting of outcomes
☒	☐	Estimates of effect sizes (e.g. Cohen's *d*, Pearson's *r*), indicating how they were calculated

Our web collection on statistics for biologists contains articles on many of the points above.

Software and code

Policy information about availability of computer code

Data collection	Next-generation sequencing data was collected by Illumina HiSeq 1500 (RNA-seq: single-read mode). Phase contrast imaging was performed on a Zeiss Axio Imager M1 microscope equipped with EC Plan Neofluar 100x/ 1.3 Oil Ph3 objective (Zeiss). Confocal Microscopy: Images were acquired with an Olympus 100x objective with numerical aperture of 1.35, using a Yokogawa spinning disk confocal scanner and laser excitation at 488 nm.
Data analysis	Cell centerline curvature, cell length, and cell area of V. cholerae cells were determined by using the ImageJ plug-in MicrobeJ v5.13k.<br>Biofilm images were analysed with BiofilmQ software available from Drescher Lab (https://www.biorxiv.org/content/10.1101/735423v1).<br>3-D cell rendering was done using ParaView software (version 5.6.0-RC2; https://www.paraview.org/).<br>Biofilm images were prepared with NIS-Elements AR Analysis software (version 4.50.000; Nikon).<br>Band intensities of Western blots were quantified using the BIO-1D software (version 15.08; Vilber).<br>Band intensities of Northern blots were quantified using the GelQuant software (version 1.8.2; BiochemLabSolutions.com).<br>The MultAlin algorithm (version 5.4.1; http://multalin.toulouse.inra.fr/multalin/) was used to align sequences.<br>The RNAhybrid algorithm (version 2.1.2; https://bibiserv.cebitec.uni-bielefeld.de/rnahybrid) was used to predict sRNA-mRNA base-pairings.<br>Statistics were calculated using Graphpad Prism (version 8.4.3; Graphpad Software) and SigmaPlot (version 14; Systat Software)

For manuscripts utilizing custom algorithms or software that are central to the research but not yet described in published literature, software must be made available to editors/reviewers. We strongly encourage code deposition in a community repository (e.g. GitHub). See the Nature Research guidelines for submitting code & software for further information.

Data

Policy information about availability of data

All manuscripts must include a data availability statement. This statement should provide the following information, where applicable:

- Accession codes, unique identifiers, or web links for publicly available datasets
- A list of figures that have associated raw data
- A description of any restrictions on data availability

Source data to reproduce the figures presented in this manuscript are provided. The raw data of the VxrB-HIS ChIP experiment conducted by Shin et al. was obtained from Gene Expression Omnibus (GEO) under the accession number GSE135009.
Microscopy images are available on request.
The raw data of the transcriptome analyses are available at the National Center for Biotechnology Information Gene Expression Omnibus (GEO) under the accession number GSE145764.

Field-specific reporting

Please select the one below that is the best fit for your research. If you are not sure, read the appropriate sections before making your selection.

☒ Life sciences ☐ Behavioural & social sciences ☐ Ecological, evolutionary & environmental sciences

For a reference copy of the document with all sections, see nature.com/documents/nr-reporting-summary-flat.pdf

Life sciences study design

All studies must disclose on these points even when the disclosure is negative.

Sample size	All relevant information is provided in the mansucript and supplemental files
Data exclusions	No data were excluded.
Replication	The experiments were conducted in several biologically independent replicates, specified in each case in the relevant figure legend.
Randomization	Not applicable.
Blinding	Not applicable.

Reporting for specific materials, systems and methods

We require information from authors about some types of materials, experimental systems and methods used in many studies. Here, indicate whether each material, system or method listed is relevant to your study. If you are not sure if a list item applies to your research, read the appropriate section before selecting a response.

Materials & experimental systems

n/a	Involved in the study	
☐	☒	Antibodies
☒	☐	Eukaryotic cell lines
☒	☐	Palaeontology
☒	☐	Animals and other organisms
☒	☐	Human research participants
☒	☐	Clinical data

Methods

n/a	Involved in the study	
☒	☐	ChIP-seq
☒	☐	Flow cytometry
☒	☐	MRI-based neuroimaging

Antibodies

Antibodies used	- anti-FLAG antibody (Sigma, #F1804), 1:1,000 dilution in TBS-T + 3 % BSA<br>- anti-RnaPα antibody (BioLegend, #WP003), 1:5,000 dilution in TBS-T + 3 % BSA
Validation	Information about the anti-FLAG antibody can be found on the manufactor's webpage: https://www.sigmaaldrich.com/catalog/product/sigma/f1804?lang=de&region=DE&gclid=CjwKCAiAv4n9BRA9EiwA30WND9c9YIGDUY2-0pos_HK_M1sLfjEkixsUB1Z40n2xTwq2CRH4luosKxoCzGYQAvD_BwE<br>anti-RnaPα antibody: https://www.biolegend.com/en-us/products/purified-anti-e-coli-rna-polymerase-alpha-antibody-14680
